# Molecular conformation engineering in central 8*π*-electron system toward unique aggregation-induced ultra-narrowband emission with a FWHM of 13 nm

**DOI:** 10.1038/s41377-026-02277-7

**Published:** 2026-06-10

**Authors:** Lu Liu, Han Zhang, Chenfa Xiao, Bingzhu Ma, Baoxi Li, Xin He, Guanjun Xiao, Bo Zou, Baolei Tang, Hongyu Zhang, Jacky W. Y. Lam, Ben Zhong Tang, Zhiming Wang

**Affiliations:** 1 State Key Laboratory of Luminescent Materials and Devices, Guangdong Provincial Key Laboratory of Luminescence from Molecular Aggregates, South China University of Technology, Guangzhou, 510640 China; 2 Department of Chemistry, Hong Kong Branch of Chinese National Engineering Research Center for Tissue Restoration and Reconstruction, The Hong Kong University of Science and Technology, Clear Water Bay, Kowloon, 999077 Hong Kong China; 3 College of Physics, Jilin University, Changchun, 130012 China; 4 College of Chemistry, Jilin University, Changchun, 130012 China; 5 Guangdong Basic Research Center of Excellence for Aggregate Science, School of Science and Engineering, The Chinese University of Hong Kong, Shenzhen, Guangdong 518172 China

**Keywords:** Organic LEDs, Displays

## Abstract

Organic emitters with narrowband emission characteristics demonstrate significant advantages and application potential in ultra-high-definition displays and multiplexed fluorescence imaging. However, the obstinate spectral broadening phenomenon upon aggregation limits the practical application of organic narrowband emissive materials. Here, a referable strategy on conformation engineering is proposed in a central 8*π*-electron system with narrowband emissions in both solution and aggregate. Owing to the synergistic effect of strain-release-driven conformational planarity in ground state and excited-state aromaticity-driven planarity, PDBP-*b,i* exhibits minimal structural change between the S_0_ and S_1_ states, resulting in narrowband emission in solution with a full width at half maximum (FWHM) of 35 nm/0.24 eV. While in aggregate, the cross dipole stacking mode further confines molecular conformation and restricts high-frequency vibration, additionally narrowing with a record-small FWHM of 13 nm/0.08 eV. The concept of aggregation-induced ultra-narrowband emission is proposed, which also offers advantages in electroluminescence to exhibit a small FWHM of 13 nm and remaining stable as the concentration increases. These results provide new insights and instructive guidance for the design of organic narrowband emitters.

## Abstract (web summary)

The concept of aggregation-induced ultra-narrowband emission (AIUNE) was developed based on a central 8π-electron organic emitter, achieving the narrowest spectral FWHM of 13 nm reported for organic aggregates.

## Graphical abstract

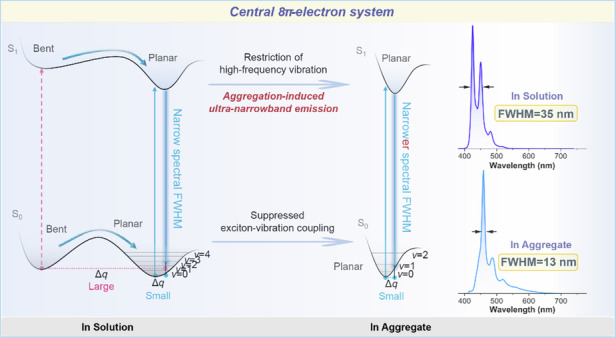

## Introduction

Organic emitters, owing to their structural diversity and tunable photophysical properties, are widely applied in optoelectronic devices, bioimaging, anti-counterfeiting, and chemical sensing^1–7^. Finely tailoring and modifying molecular structures by extending or shortening *π*-conjugated systems, along with introducing electron donors and/or acceptors, has become a common strategy for customizing specific photophysical properties^8–13^. However, this experience-based molecular design cannot explain the causes of certain intriguing luminescence phenomena or photophysical variations. For example, our group reported a series of closed-form rhodamine derivatives that exhibit strong aggregate emission in the visible region, despite the absence of identifiable conjugated chromophores. The intrinsic cause of their unique aggregation-induced emission (AIE) phenomenon is an aggregation-induced molecular conformation change, which triggers intramolecular charge transfer emission^14,15^. Another typical example is the vibration-induced emission (VIE) phenomenon reported based on dihydrophenazine derivatives, where a single molecule can exhibit multi-color emission with distinct emission peaks ranging from blue to red in response to changes in environmental conditions (e.g. temperature/viscosity)^16–18^. These significant luminescent color changes depend on continuous conformational changes in the lowest-lying excited state (S_1_). Therefore, regulating the molecular conformations of organic emitters in both the ground state (S_0_) and excited state has become an important approach for understanding and developing novel photophysical phenomena.

**Fig. 1:**
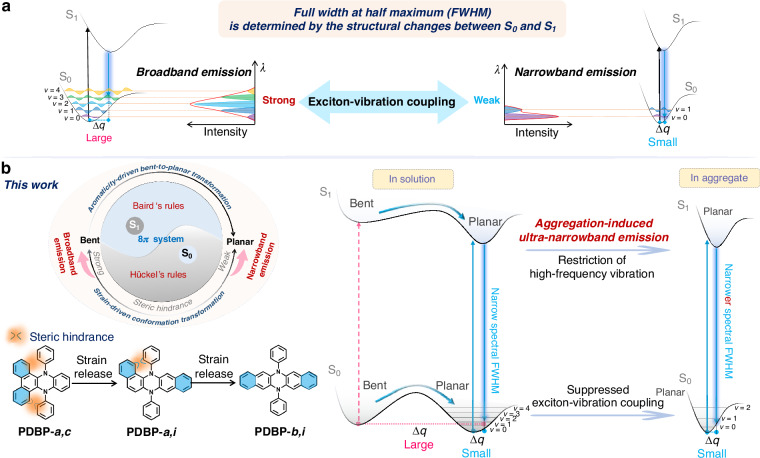
Schematic illustration of FWHM determine mechanism. **a** Mechanism of narrowband emission. **b** Left: Spectral modulating mechanism of molecules with 8*π*-electron structures and chemical structures of PDBP-*a,c*, PDBP-*a,i*, and PDBP-*b,i*; Right: a schematic representation of photophysical and conformation transformation process in this work

The emerging narrowband emission characteristics of organic emitters have attracted increasing research attention in recent years^19–24^. In general, the spectral FWHM is determined by the structural changes between the S_0_ and S_1_ states (Fig. 1a)^25,26^. The larger the structural changes between the S_0_ and S_1_ states, the more vibronic transitions (0–1, 0–2, 0–3, etc.) are generated through strong coupling with excitons, leading to a broader emission spectrum. Conversely, when the structural changes between the S_0_ and S_1_ states are minimized, the suppression of exciton-vibration coupling results in a narrower and sharper emission spectrum. The most representative material system is the 1,4-BN-heteroarene with multiple resonance (MR) effect^27–31^. Its planar and rigid structural framework and non-bonding characteristics effectively suppress low-frequency vibrations such as rotation and bending, as well as high-frequency bond stretching vibrations, enabling the FWHM of organic emitters to be less than 30 nm (<0.15 eV). However, this type of narrowband emitter, due to its large planar conjugated structures, tends to generate excimers through *π–π* stacking, resulting in significant spectral broadening in the aggregated state^32,33^. One solution is to use host-guest doping systems with ultra-low concentrations (≤2 wt%) to preserve the narrowband spectral characteristics of the single-molecule state as much as possible, but this also increases the precision requirements of the vapor deposition process^34^. This raises the question: can organic emitters with narrowband emission at the molecular level maintain a narrowband spectrum after aggregation, or even become further narrowed?

There are more and more cases indicating that the properties of materials are not solely determined by molecular structure, and the multibody interactions within the mesoscopic structures of aggregates also greatly influence their macroscopic performance^35–40^. Previous studies have reported the spectral narrowing phenomenon in organic emitters upon aggregation. For example, Tian et al. reported a series of butterfly-like molecules exhibiting cross dipole stacking or herringbone packing modes in crystals^41–43^. Due to the fixation of their molecular conformations by multiple intermolecular interactions, the torsional and vibrational motions of the molecules are markedly inhibited, resulting in spectral narrowing of the emission in the crystals. Based on carbazole-pyrimidine isomers, Yan et al. revealed that strengthened steric hindrance and multiple non-covalent interactions induce their narrowband emission in the aggregated state^44^. However, the spectral FWHMs of these organic emitters in aggregate remain relatively large (>0.25 eV), which may be related to their broadband emission spectra at the molecular level. Therefore, achieving narrowband emission in aggregate requires consideration at both the molecular and aggregate levels.

In polycyclic *π*-conjugated molecules with an 8*π*-electron central ring, the interplay of *Hückel* and *Baird* aromaticity can drive the conformational change in the S_0_ and the S_1_ states, respectively (Fig. 1b)^45–47^. Meanwhile, in this type of structure, multiple intermolecular C−H···*π* interactions are generated in the aggregated state, thereby inducing a variety of aggregation effects^48^. These structural features at both the molecular and aggregate levels make it an ideal model for regulating the shape of the emission spectrum. PDBP-*a,c* is a representative dihydrophenazine derivative exhibiting a large Stokes shift and multiple emission characteristics^15,46^. This behavior is attributed to the steric hindrance between its *N*,*N*’-substituted phenyl rings and phenanthrene rings, which perturbs the potential energy surface of the S_1_ state, leading to multiple conformational emissive states.

Building on the aforementioned mechanism of narrowband emission, we selected PDBP-*a,c* as a reference molecule, by gradually eliminating the steric hindrance effects in its structure, designed and synthesized two isomers, PDBP-*a,i* and PDBP-*b,i*, with the aim of regulating the structural changes between the S_0_ and S_1_ state (Fig. 1b). At S_0_, the gradually eliminated steric hindrance drives the strain release to undergo bent-to-planar conformational transformation. At S_1_, the aromaticity conversion also drives bent-to-planar conformational transformation. Therefore, the conversion from ultra-broadband FWHM of 116 nm to ultra-narrowband FWHM of 35 nm in the isomers were achieved. PDBP-*b,i* shows negligible conformation evolution with an extremely small structural difference between its planar conformations in both S_0_ and S_1_ states, corresponding to a root mean square deviation (RMSD) of only 0.01 Å. In crystals, PDBP-*b,i* adopts a cross dipole stacking mode, where multiple C−H···*π* interactions are present, and no significant *π*−*π* interactions are observed. Such aggregation mode can restrict the high-frequency vibration to suppressing exciton-vibration coupling for further narrowing the emission with FWHM of 13 nm. To the best of our knowledge, 13 nm represents the smallest FWHM in homogeneous aggregate among organic emitters of all types. More importantly, the electroluminescence (EL) spectra of the deep-blue organic light-emitting diodes (OLEDs) based on PDBP-*b,i* also show an FWHM of merely 13 nm, which is comparable to the smallest FWHM reported for OLEDs based on MR emitters^19,32,49–74^. Even at high doping concentrations, the spectral FWHM remains stable.

## Results and discussion

### Synthesis and characterization

The synthetic routes of PDBP-*a,c*, PDBP-*a,i*, and PDBP-*b,i* are shown in Supplementary Scheme S1. PDPB-*a*,*c* was synthesized according to the reported procedure^15^. For PDBP-*a*,*i* and PDBP-*b*,*i*, the key intermediates, DHPDBP-*a,i* and DHPDBP-*b,i*, were first synthesized *via* a solid-state cyclization reaction of *o*-diaminobenzene derivatives and *o*-diaminophenyl derivatives, respectively. These intermediates subsequently underwent Buchwald-Hartwig coupling with bromobenzene to yield the target compounds PDBP-*a*,*i* and PDBP-*b*,*i*. The molecular structures of PDBP-*a*,*c*, PDBP-*a*,*i*, and PDBP-*b*,*i* were well confirmed by NMR and high-resolution mass spectrometry analyses (Supplementary Figs. S1−S5).

### Photophysical properties in solution

**Fig. 2:**
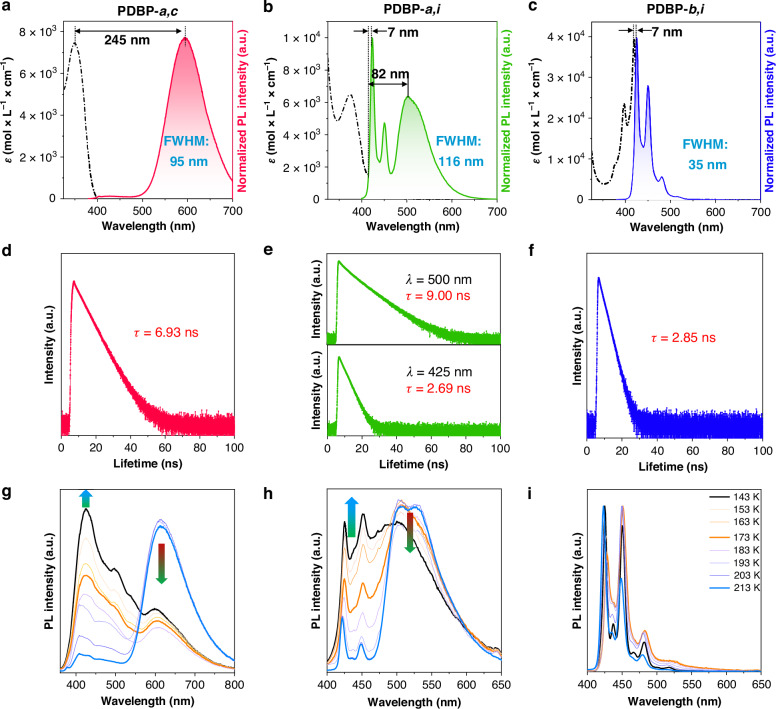
Photophysical properties of PDBP-*a,c*, PDBP-*a,i*, and PDBP-*b,i*. UV-vis and PL spectra of **a** PDBP-*a,c*, **b** PDBP-*a,i* and **c** PDBP-*b,i* in toluene solution (10^−5^ M). Transient PL decay curves of **d** PDBP-*a,c*, **e** PDBP-*a,i* and **f** PDBP-*b,i*. Temperature-dependent PL spectra of **g** PDBP-*a,c*, **h** PDBP-*a,i* and **i** PDBP-*b,i* in *n*-butanol from 143 K to 213 K with various viscosities

Initially, the UV-vis absorption and photoluminescent (PL) spectra of PDBP-*a*,*c*, PDBP-*a*,*i*, and PDBP-*b*,*i* were measured in toluene solution (10^−5^ M) (Fig. 2a−c and Supplementary Table S1). Stemming from significant structural relaxation in the excited state, PDBP-*a,c* exhibits a pronounced Stokes shift of 245 nm, with the corresponding first absorption maximum at 350 nm and PL peak at 595 nm, respectively^15^. Additionally, a large FWHM value of 95 nm is recorded for the PL spectrum of PDBP-*a,c*, indicating considerable structural changes between the S_0_ and S_1_ states. In contrast to PDBP-*a,c*, the first absorption maximum of PDBP-*a,i* red-shifts to 418 nm, likely due to the extension of *π*-conjugation and an increase in the effective conjugation path by eliminating the steric hindrance between the side benzene ring and the phenanthrene ring. Intriguingly, PDBP-*a,i* exhibits dual-emission characteristics, featuring a short-wavelength band at 425 nm with hyperfine vibronic structures and a structureless long-wavelength band at 500 nm, suggesting the presence of two stable conformations in its S_1_ state. The corresponding Stokes shift were measured at 7 nm and 82 nm, respectively. For PDBP-*b,i*, only a pair of mirror-image absorbance and emission spectra with hyperfine vibronic structures was observed, with the corresponding first absorption maximum, PL peak, and Stokes shift at 418 nm, 425 nm, and 7 nm, respectively, similar with one of the absorbance and emission pairs of PDBP-*a,i*. The molar absorptivity of PDBP-*b,i* (*ε* = 39640 mol × L^−1^ × cm^−1^) at 418 nm far exceeds that of PDBP-*a,i* (*ε* = 1569 mol × L^−1^ × cm^−1^), which may be attributed to their different degrees of *π*-conjugation. Remarkably, PDBP-*b,i* achieves a high color purity deep-blue emission peaking at 425 nm, with a small FWHM of 35 nm/0.24 eV and CIE coordinates of (0.156, 0.032). The PL quantum yield (PLQY) of PDBP-*a,c*, PDBP-*a,i*, and PDBP-*b,i* in toluene solution is recorded as 22%, 48%, and 75%, respectively.

To investigate the excited state properties of PDBP-*a,i* and PDBP-*b,i*, their PL spectra were measured in solvents of varying polarity (Supplementary Fig. S6 and Table S2). As the solvent polarity increases from hexane to acetonitrile, both the short-wavelength band in the PL spectrum of PDBP-*a,i* and the PL spectrum of PDBP-*b,i* show only a minor red-shift, with no significant change in their hyperfine vibronic structures, indicating a typical locally excited (LE)-dominant state. By contrast, the long-wavelength band in the PL spectrum of PDBP-*a,i* exhibits a 28 nm red-shift accompanied by spectral broadening, which can be attributed to a charge transfer (CT)-dominant state. Therefore, compared to PDBP-*b,i*, the lower PLQY of PDBP-*a,c* and PDBP-*a,i* in solution is likely due to greater energy consumption during the excited-state relaxation process and enhanced non-radiative decay from the CT state. The transient PL decay curves of PDBP-*a,c*, PDBP-*a,i*, and PDBP-*b,i* were further measured in toluene solution (Fig. 2d–f). The fluorescence lifetimes for PDBP-*a,c* and PDBP-*b*,*i* were recorded as 6.93 ns and 2.85 ns, respectively. By comparison, PDBP-*a,i* shows two distinct lifetimes of 2.69 ns at 425 nm and 9.00 ns at 500 nm, which further confirms that the dual-emission behavior arises from two distinct excited-state conformations with different photophysical properties.

Previous studies have shown that although PDBP-*a,c* exhibits a single-peak emission at room temperature, multiple distinct local minima during excited-state structural relaxation can be stabilized by lowering the temperature and increasing the viscosity of *n*-butanol, resulting in multi-color emission behavior (Fig. 2g)^15^. Based on this method, we recorded the temperature-dependent fluorescence spectra of PDBP-*a,i* and PDBP-*b,i* in *n*-butanol from 213 to 143 K with various viscosities (Fig. 2h, i). As the temperature decreases, the dual-emission characteristic of PDBP-*a,i* is consistently maintained, accompanied by an increase in the intensity of the short-wavelength band. These results provide strong evidence that two stable conformations can be stabilized during its excited-state structural evolution at room temperature. Similarly, the single-peak emission characteristic of PDBP-*b,i* is maintained at low temperatures, with no new peaks appearing, indicating that its excited-state structural evolution involves only one stable conformation. To further elucidate the composition of the excited states, nanosecond time-resolved PL spectra were measured at room temperature in toluene (Supplementary Fig. S7). Upon excitation, PDBP-*a,i* exhibits a dual-emission characteristic. As time elapsed, the short-wavelength band decays more rapidly than the long-wavelength band, indicating that they originate from different excited-state conformations. In contrast, during the decay process, PDBP-*b*,*i* exhibits only a single-peak emission, indicating that it exists in a single excited-state conformation.

### Mechanism study of narrowband emission in solution

**Fig. 3:**
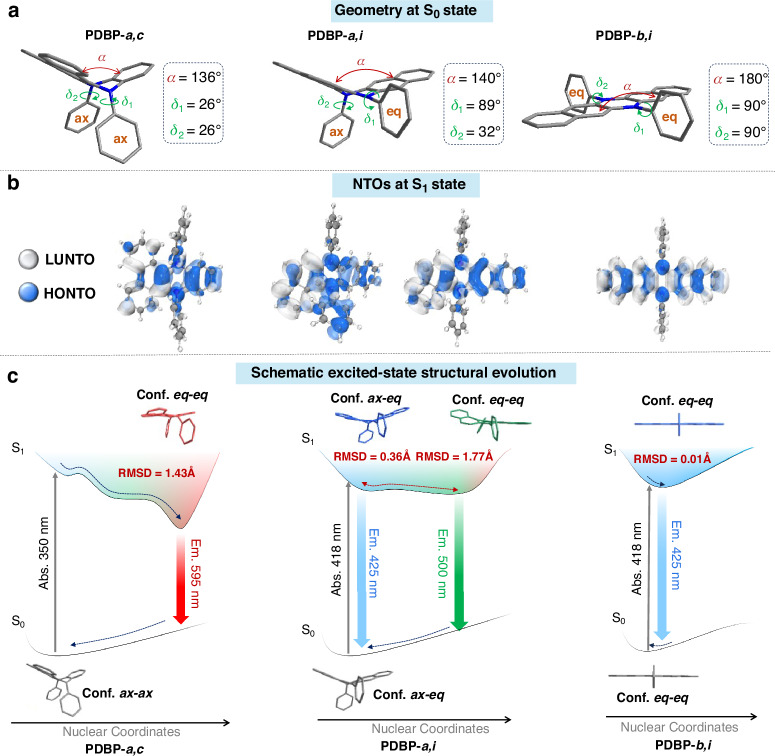
Molecular simulation of PDBP-*a,c*, PDBP-*a,i* and PDBP-*b,i.* **a** Optimized S_0_ geometry structures and **b** NTOs of S_1_ → S_0_ for PDBP-*a,c*, PDBP-*a,i*, and PDBP-*b,i*. **c** Schematic potential energy surfaces for the structural evolution of PDBP-*a,c*, PDBP-*a,i*, and PDBP-*b,i*

The ground-state geometries of PDBP-*a,c*, PDBP-*a,i*, and PDBP-*b,i* in the S_0_ state were calculated using density functional theory (DFT) simulations (Fig. 3a, Supplementary Figs. S8−S10). The strong steric hindrance between the *N,N*′-substituted phenyl rings and phenanthrene rings forces PDBP-*a,c* to bend along the *N*···*N* axis, forming a V-shaped structure with a bent angle (*α*) of 136°. The two *N*-substituted phenyl rings are nearly parallel to the dihydrophenazine core, with torsion angles (*δ*_1_ and *δ*_2_) of 26°, corresponding to a quasi-*axial*/quasi-*axial* (*ax-ax*) conformation. These results are agreement with those reported in the literature^15,46,75,76^. With half of the steric hindrance eliminated, the structural strain in PDBP-*a,i* is released, resulting in a decrease in the degree of bent angle (*α* = 140°) and a trend toward planarization. Meanwhile, one of the *N*-substituted phenyl rings rotates nearly orthogonally to the dihydrophenazine core with a torsion angle *δ*_1_ of 89°, leading to a quasi-axial/quasi-equational (*ax-eq*) conformation in PDBP-*a,i*. Due to the complete elimination of steric hindrance, PDBP-*b,i* exhibits a strain-free planar structure with an *α* of 180°, while both *N,N*′-substituted phenyl rings adopt an equatorial-equatorial (*eq*-*eq*) conformation. The strain-release-driven planarization observed in these three molecules confirms the feasibility of the design for regulating the ground-state conformation through the elimination of steric hindrance.

Considering the versatile exited-state structural evolution of dihydrophenazine derivatives^16^, we performed potential energy surface (PES) simulations by scanning *α* of the dihydrophenazine core from 130° to 180° for PDBP-*a,i*, and PDBP-*b,i* in the S_1_ state (Supplementary Fig. S11). According to Baird’s rule^77,78^, the 8*π*-electron central ring in dihydrophenazine derivative prefers planarization in the S_1_ state due to excited-state aromaticity^17^. As demonstrated by the nuclear independent chemical shift (NICS) calculations, PDBP-*a,c*, PDBP-*a,i* and PDBP-*b,i* undergo an inversion from antiaromaticity to aromaticity when transition from the ground to the excited state (Supplementary Fig. S12). As a result, the structural relaxation toward planarity (*α* = 180°) is thermodynamically favorable for these compounds. Meanwhile, the concomitant structural relaxation from the *N*,*N’*-substituted phenyl rings changes as steric hindrance is gradually eliminated, leading to reduced perturbation of the S_1_ PES, which results in different distributions of global and local energy minima for PDBP-*a,c*, PDBP-*a,i*, and PDBP-*b,i*. The S_1_ PES of PBDP-*a,i* shows a global energy minimum and a local energy minimum, which is coincidence with the reported PDBP-*a,c*^15^. In contrast, PDBP-*b,i* has only a global energy minimum, thus exhibiting single-peak emission at both room and low temperatures. As reported^15^, the global energy minimum of PDBP-*a,c* is located at *α* = 161°, with the corresponding S_1_ structure exhibiting an *eq*-*eq* conformation (Supplementary Fig. S13). The vertical emission for the S_1_ → S_0_ transition is calculated to be 561 nm, which corresponds to the experimental emission at 595 nm (Supplementary Table S3). For PDBP-*a,i*, the global and local energy minima are located at *α* = 144° and *α* = 171°, with the corresponding S_1_ structures exhibiting *ax*-*eq* and *eq*-*eq* conformations, respectively (Supplementary Figs. S14 and S15). The calculated vertical emissions are 473 nm and 561 nm, which correspond to the experimental emissions at 425 nm and 500 nm (Supplementary Table S3). The global energy minimum of PDBP-*b,i* is located at *α* = 180°, with the corresponding S_1_ structure exhibiting an *eq*-*eq* conformation (Supplementary Fig. S16). The vertical emission is calculated to be 427 nm, which corresponds to the experimental emission at 425 nm. Further natural transition orbital (NTO) analyses were conducted on the emissive-state conformations of these three molecules (Fig. 3b). The *eq*-*eq* conformation of PDBP-*a,c* shows spatial separation of holes and electrons, which corresponds to a CT-dominant characteristic. In contrast, the *eq*-*eq* conformation of PDBP-*b,i* exhibits an LE-dominant characteristic, with nearly full overlap between the hole and electron. These findings are agreement with the structureless and hyperfine vibrational features observed in the experimental spectra. For PDBP-*a,i*, the *ax*-*eq* conformation shows an LE-dominant characteristic, while the *eq*-*eq* conformation shows a CT-dominant characteristic, which is consistent with its dual-emission characteristics.

**Fig. 4:**
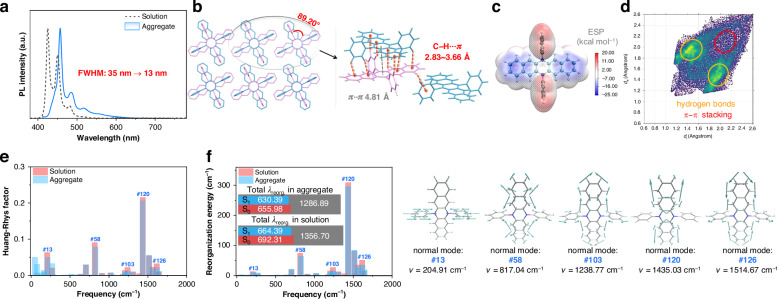
Photophysical and crystallographic properties in aggregates. **a** PL spectra of PDBP-*b,i* in solution and solid powders. Structural information for **b** PDBP-*b,i* in crystal (Left: Packing structures; Right: Intermolecular interaction. H-atoms were omitted for clear). **c** ESP surface of PDBP-*b,i* ranging from 20 to −20 kcal mol^−1^, where positive one represents electron-rich and negative one represents electron-deficient. **d** Intermolecular interactions with 2D fingerprint plots defined by (*d*_*i*_, *d*_*e*_) pairs. The blue color stands for a small number of points and the yellow stands for a large number of points. **e** Huang-Rhys factors and **f** reorganization energies S_1_ → S_0_ of PDBP-*b,i* in solution and aggregates (right: vector of vibration corresponding to the normal mode)

Since the spectral FWHM is determined by the structural changes between the S_0_ and S_1_ states, we calculated the RMSD between the S_0_ and S_1_ conformations of these three molecules (Fig. 3c and Supplementary Fig. S17). PDBP-*a,c* undergoes significant structural changes from the *ax*-*ax* conformation in the S_0_ state to the *eq*-*eq* conformation in the S_1_ state, resulting in an RMSD of 1.43 Å. Consequently, the spectrum of PDBP-*a,c* in solution displays a large FWHM. For PDBP-*a,i*, the structural change from the *ax*-*eq* conformation in the S_0_ state to the *ax*-*eq* conformation in the S_1_ state is minimal, with the RMSD significantly reduced to 0.36 Å. However, the transformation from the *ax*-*eq* conformation in the S_0_ state to the *eq*-*eq* conformation in the S_1_ state involves significant structural changes, resulting in a large RMSD of 1.77 Å. Therefore, the spectrum of PDBP-*a,i* features both a narrow short-wavelength band and a broad long-wavelength band. In particularly, both the S_0_ and S_1_ states of PDBP-*b,i* exhibit planar *eq*-*eq* conformations, with a minimal structural difference, as indicated by an RMSD of only 0.01 Å. The synergistic effect of strain-release-driven planarization in the S_0_ state and excited-state aromaticity-driven planarization in the S_1_ state ultimately results in its narrowband emission in solution. Furthermore, to gain deeper insights into the spectral progression, the Huang−Rhys factors (*S*) (Fig. 4e) and reorganization energy (*λ*_reorg)_ (Fig. 4f) for the different vibrational modes of PDBP-*b,i* were determined. Only a few vibrational modes are observed, indicating that the exciton-vibration coupling is significantly suppressed, which leads to a narrowband spectrum in solution^79^. The dominant vibrational modes of PDBP-*b,i* are located at frequencies of 204.91, 817.04, and 1435.03 cm^−1^, corresponding to bending vibration of the *N,N*′-substituted phenyl rings, bending vibration of the dihydrophenazine, and stretching vibration of the *π*-conjugated skeleton^80^. These vibrational modes contribute to the hyperfine vibrational structure in the spectrum of PDBP-*b,i*. Furthermore, the primary vibrational modes involved in electron-vibration coupling, which affect the spectral peak shape, have been verified in the vibrationally resolved electronic spectrum (Supplementary Fig. S18).

### The phenomenon of aggregation-induced ultra-narrowband emission

The photophysical properties of PDBP-*a,i* and PDBP-*b,i* were measured in solid powders and are summarized in Supplementary Table S4. PDBP-*a,i* still exhibits dual-emission characteristics in solid powders, with emission peaks at 460 nm and 500 nm, respectively (Supplementary Fig. S19). The corresponding fluorescence lifetimes were recorded as 1.18 ns and 14.85 ns (Supplementary Fig. S20), suggesting that its excited state retains two stable conformations with distinct characteristics. Compared to the solution state, the spectral FWHM in solid powders narrowed from 116 nm to 111 nm. Excitingly, PDBP-*b,i* exhibits a rare aggregation-induced ultra-narrowband emission (AIUNE) phenomenon, with an emission peak at 460 nm and a FWHM of merely 13 nm/0.08 eV (Fig. 4a). To the best of our knowledge, 13 nm represents the smallest FWHM reported in a homogeneous aggregated state among all types of organic emitters.

To investigate the cause of spectral narrowing upon aggregation, the single-crystal structures of PDBP-*a,i* (CCDC: 2444441) and PDBP-*b,i* (CCDC: 2444438) were obtained *via* slow solvent evaporation and are shown in Supplementary Fig. S16 and Fig. 4b. In PDBP-*a,i*, the monomer adopts an *ax*-*eq* conformation (Supplementary Fig. S21), where steric hindrance from the *N,N*′-substituted phenyl rings prevent *π*−*π* stacking. Instead, abundant C−H···*π* interactions are observed between the *N,N*′-substituted phenyl rings and the neighboring dihydrophenazine core, with distances of 2.80–3.64 Å (Fig. 4b). These multiple weak interactions drive PDBP-*a,i* to form a herringbone-packed structure along *b*-axis with *θ* = 73.42° (Supplementary Fig. S22)^41,81,82^. In PDBP-*b,i*, the monomer shows an *eq*-*eq* conformation (Supplementary Fig. S23), where increased steric hindrance from the lateral *N,N*′-substituted phenyl rings inhibit face-to-face *π*−*π* stacking and the vertical distance between the molecular planes is 4.81 Å (Fig. 4b). Likewise, multiple C−H···*π* interactions are favored between the *N,N*′-substituted phenyl rings and the neighboring dihydrophenazine core (2.83–3.66 Å), inducing the molecules to form a cross dipole stacking mode with *θ* = 89.20° along the *c*-axis (Fig. 4b)^42,43,83^. According to previous studies, these stacking modes facilitate spectral narrowing by inhibiting the torsional and vibrational motions of the molecules^41,43^.

To investigate the impact of molecular structure on aggregate stacking, the electrostatic potential (ESP) surface of PDBP-*b,i* molecule was simulated (Fig. 4c). The luminescent dibenzo[*b,i*]phenazine skeleton and the substituted phenyl groups have distinct negative and positive electrostatic potentials, which may account for the crossed dipole stacking through the inter-attraction between the electronegative parts and electropositive parts. Furthermore, the phenyl groups are orthogonal to the dibenzo[*b*,*i*]phenazine skeleton, introducing significant steric hindrance that prevents excimer formation. Hirshfeld surface analyses of PDBP-*b,i* disclose that strong C−H∙∙∙*π* interactions between the phenyl groups and the dibenzo[*b*,*i*]phenazine core, while *π*−*π* interactions are negligible (Fig. 4d and Supplementary Fig. S24). These finding suggest that molecular structures with uniform electronegativity, along with introduction of non-conjugated steric hindrance groups with opposing electronegatively, are beneficial for enlarge the distance between luminescent centers. To further elucidate the influence of different intermolecular interactions on the spectral shape, the PL spectra of PDBP-*b,i* solid powder were measured under increased isotropic hydrostatic pressures (Supplementary Fig. S25). Under hydrostatic pressures below 0.5 GPa, the spectral FWHM shows almost no change with increasing pressure, accompanied by a decrease in the shoulder peak. As the pressure continues to rise, the spectra exhibit a sustained redshift and broadening, with the formation of excimer emission observed in the long-wavelength range. This is because the face-to-face intermolecular distance of PDBP-*b,i* decreases with increasing isotropic hydrostatic pressure. At lower pressures, a moderate reduction in intermolecular distance enhances C−H···*π* interactions without inducing *π*−*π* stacking; however, with further pressure increase, the continued reduction in intermolecular distance promotes the occurrence of *π*−*π* interactions. PDBP-*b,i* already exhibits a small spectral FWHM in solution, and after aggregation, no excimer emission is generated due to absent *π*−*π* interactions. Furthermore, the presence of multiple weak C−H···*π* interactions restricts changes in molecular conformation, thereby displaying an AIUNE characteristic.

For deeper understanding the mechanism of AIUNE property, *S* and *λ*_reorg_ of PDBP-*b,i* in solution and aggregate are compared in Fig. 4e, f. The dominant vibrational mode around 204.91, 817.04, 1238.77, 1435.03, and 1514.67 cm^−1^ are suppressed from solution to aggregate, indicating the aggregation effect of PDBP-*b,i* can suppress electron-vibration coupling. Meanwhile, the total *λ*_reorg_ at S_0_ and S_1_ states reduces from 692.31 and 664.39 cm^−1^ in solution to 655.98 and 630.39 cm^−1^ in aggregate, which further confirms that aggregation effect can confine the structural change of both S_0_ and S_1_ states. These results demonstrate that the AIUNE property, arising from restricted intramolecular vibrations, reduces the Franck-Condon factors between vibrational wavefunctions during electronic transitions, resulting in a narrower spectral FWHM. (Fig. 4e). Notably, the relationship between *λ*_reorg_ and the normal vibrational modes illustrates that the high-frequency vibrations (1000–1650 cm^−1^) contribute more substantially to reorganization energy reduction than that in the low-frequency region (0–1000 cm^−1^) (Fig. 4f). The suppression of high-frequency vibrations manifests as decreased intensity of the spectral shoulder peak, consistent with experimental observations (Fig. 4a). Therefore, we attribute the AIUNE mechanism in PDBP-*b,i* to aggregation-induced restriction of high-frequency vibrations.

### OLED applications

**Fig. 5:**
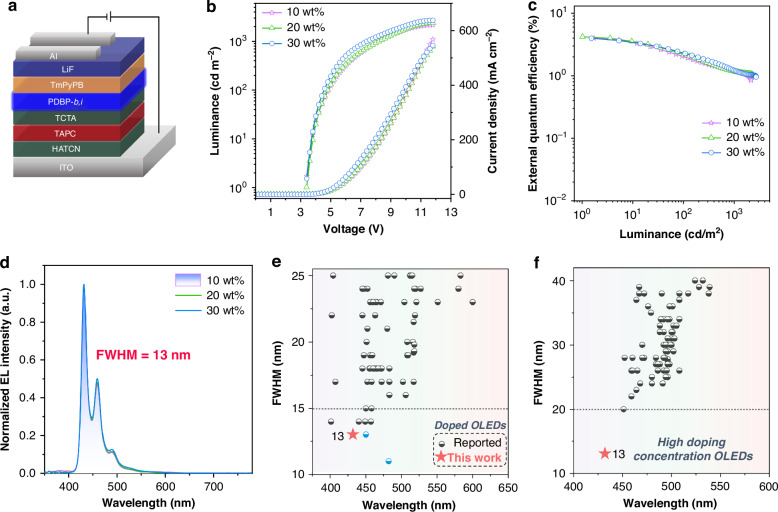
OLED performances based on PDBP-*b,i*. **a** Device configuration of the OLEDs. **b** *J-V-L*, **c** EQE-*L* curves and **d** EL spectra of OLEDs based on PDBP-*b,i*. Summary of the FWHM values of reported **e** doped OLEDs and **f** high doping concentration (>10 wt%) OLEDs

The narrowband emission characteristics of PDBP-*b,i* inspired us to evaluate its electroluminescent properties. We first performed the thermogravimetric analysis (TGA) and differential scanning calorimetry (DSC) measurements (Supplementary Fig. S26). The decomposition temperature (*T*_d_, corresponding to 5% weight loss) of 352 °C, indicative of its suitability for vacuum deposition. The glass transition temperatures (*T*_g_) of PDBP-*b,i* is about 232 °C. The energy levels of the HOMO and LUMO were estimated through cyclic voltammetry (CV) measurement (Supplementary Fig. S27), which are −4.7 and −1.8 eV, respectively. Subsequently, OLEDs were fabricated with the optimized configuration of ITO/hexaazatriphenylenehexacabonitrile (HATCN, 5 nm, hole-injecting layer)/1,10-bis(di-4-tolylaminophenyl)cyclohexane (TAPC, 40 nm, hole-transporting layer)/tris(4-carbazoyl-9-ylphenyl)amine (TCTA, 5 nm, hole-buffer layer)/EML (20 nm, emitting layer)/1,3,5-tri(m-pyrid-3-yl-phenyl)benzene (TmPyPB, 40 nm, electron-transporting layer)/LiF (1 nm, electron-injecting layer)/Al (120 nm) (Fig. 5, Supplementary Fig. S28 and Table S5), where the doped films of PDBP-*b,i* with the doping concentrations of 10, 20, and 30 wt% in mCBP host function as EMLs. All the devices can be turned on at voltages of 3.4 V and radiate intense deep-blue EL emissions with maximum luminance (*L*_max_) over 2000 cd m^-2^. The EL spectra resemble its PL spectrum in doped films (Supplementary Fig. S29), indicative of good exciton confinement in the emitting layer. The maximum emission peak was recorded at 432 nm for the 10 wt% doped OLED based on PDBP-*b,i*, with the corresponding FWHM value of 13 nm. which is comparable to the smallest FWHM value for reported MR-based OLEDs. (Fig. 5e, f, and Supplementary Tables S6, S7) In particular, the FWHMs of the PDBP-*b,i* devices remain at 13 nm as the doping concentration increases from 10 wt% to 30 wt%, demonstrating the superiority of the molecular design with an AIUNE property. The optimal deep-blue OLED based on PDBP-*b,i* exhibits a good external quantum efficiency (EQE) of 4.2%, with CIE coordinates of (0.156, 0.045) that meets the BT.2020 blue standard. According to the fluorescence (300 K) and phosphorescence (77 K) spectra of PDBP-*b,i* in toluene (Supplementary Fig. S30), the S_1_ and T_1_ energy levels were calculated to be 2.91 and 2.53 eV, respectively, corresponding to a large Δ*E*_ST_ value of 0.48 eV. The large Δ*E*_ST_ value contributes to insufficient utilization of triplet excitons in OLEDs, resulting in less favorable EQE. Therefore, strategies such as introducing intramolecular short-range CT through structural modification and employing interlayer-sensitization^84^ *via* device engineering are expected to enhance the triple-exciton harvesting, ultimately improving EQE in future OLEDs. The roll-off in EQE at high luminance of these OLEDs should be caused by the singlet-polaron annihilation (SPA) process (Supplementary Fig. S31).

## Discussion

In summary, we successfully developed an innovative organic emitter that exhibits narrowband emission characteristics in both solution and aggregate. By comparing three dihydrophenazine isomer derivatives (PDBP-*a,c*, PDBP-*a,i*, and PDBP-*b,i*), we found that stepwise elimination of steric hindrance within the molecules not only releases structural strain, inducing a bent-to-planar conformational transformation in the S_0_ state, but also influences the progression of excited-state aromaticity-driven planarization in the S_1_ state. As a result, these molecules exhibit distinct spectral profiles. Specifically, the planar conformation of PDBP-*b,i* in both the S_0_ and S_1_ states results in minimal structural change, with a small RMSD of 0.01 Å, which gives rise to narrowband emission in solution with a peak at 425 nm and a small FWHM of 35 nm/0.24 eV. Upon aggregation, the cross-dipole stacking mode of PDBP-*b,i* confines the change of molecular conformation and restricts high-frequency vibration through multiple weak C−H···*π* interactions, leading to a further narrowing of the spectrum in the solid state, with a peak at 460 nm and a record-small FWHM of 13 nm/0.08 eV. This unique AIUNE characteristic also demonstrates superiority in OLED applications, with the EL spectra exhibiting an FWHM of only 13 nm and remaining stable as the doping concentration increases. Our achievement here not only enriches the material system of organic narrowband emitters but also provides new insights into designing organic emitters with narrowband emission characteristics in both solution and aggregate.

## Methods

### Reagents and instruments

All reagents and solvents were used as received without further purification. ^1^H and ^13^C NMR spectra were recorded on Bruker AV 400/500 MHz spectrometer, with tetramethylsilance (TMS, *δ* = 0) as the internal standard is. UV-vis absorption spectra were recorded using SHIMADZU UV-2600 absorption spectrometer. PL spectra were measured on the HORIBA Fluoromax-plus fluorescence spectrometer. The absolute fluorescence quantum yield was determined using a HAMAMATSU C11347-11 Quantaurus-QY absolute photoluminescence quantum yield measurement system. Fluorescence decay testing and nanosecond time correlated single photon counting (TCSPC) technique were carried on the spectrometer equipped with FLS1000 fluorescence spectrometer. TGA was carried on a TA TGA Q5000 under dry nitrogen at a heating rate of 10 °C min^−1^. DSC analysis was carried out on a DSC Q1000 from 30 to 300 °C under dry nitrogen at a heating rate of 10 °C min^−1^. CV were performed on a CHI 610E A14297 in a solution of tetra-*n*-butylammonium hexafluorophosphate (Bu_4_NPF_6_) (0.1 M) in dichloromethane (DCM) at a scan rate of 50 mV s^‒1^, using a platinum wire as the auxiliary electrode, a glass carbon disk as the working electrode and Ag/Ag^+^ as the reference electrode. The HOMO was approximated from the ionization potential (IP_CV_) values obtained through CV measurement. HOMO= [*E*_ox_ − *E*_1/2_(Fc/Fc^+^) + 4.8] eV, where *E*_ox_ represent the onset oxidation potential and the reduction potential relative to Fc/Fc^+^ (4.8 eV), respectively, and *E*_1/2_ (Fc/Fc^+^) represents the calibrated value. LUMO = HOMO +Δ*E*_g_. Δ*E*_g_: The optical bandgap.

### Synthesis procedures

9,14-diphenyl-9,14-dihydrodibenzo[*a,i*]phenazine (PDBP-*a,i*): To an oven-dried Schlenk flask equipped with a magnetic stirring bar, benzene-1,2-diamine (1.58 g, 10 mmol,1 equiv.) and naphthalene-1,2-diol (1.60 g, 10 mmol, 1equiv.) was added. The flask was sealed and evacuated/back-filled with nitrogen three times. The reaction mixture was heated to 200 °C with stirring condition, and was kept for 1 h. Then, the reaction was cooled down to room temperature. The resulting mixture was washed by methanol and acetone for 3 times and drying at vacuum condition for 6 h, that pale yellow powder -like product of DHDBP-*a,i* (1.33 g, 43% yield) was obtained. Then, DHDBP-*a,i* (705 mg, 2.5 mmol, 1 equiv.), Pd_2_(dba)_3_ (139 mg,0.13 mmol, 0.05 equiv.), ^*t*^Bu_3_PHBF_4_ (72 mg, 0.25 mmol, 0.1 equiv), Na^*t*^OBu (810 mg, 10 mmol, 4 equiv.) and bromobenzene (970 mg, 6.25 mmol, 2.5 equiv.) were added to an oven-dried Schlenk flask equipped with a magnetic stirring bar. The flask was sealed and evacuated/back-filled with nitrogen for three times, and 50 mL toluene was added. The reaction was heated to 120 °C with stirring condition, and was kept for 12 h. Then, the reaction was cooled down to room temperature. The resulting mixture was extracted twice with dichloromethane. The organic phase was concentrated under reduced pressure to obtain the crude product, which was purified by column chromatography and recrystallization to afford yellow powder product. 9,14-diphenyl-9,14-dihydrodibenzo[*a,i*]phenazine (PDBP-*b,i*): It was prepared according to the synthesized procedure of PDBP-*a,i*, and naphthalene-1,2-diol was replaced by naphthalene-2,3-diol.

### Computational methodology

Geometry optimization and energy calculation of the ground state (S_0_) PDBP-*a,c*, PDBP-*a,i* and PDBP-*b,i* were generated by DFT method, respectively, using B3LYP/6-311G(d,p) method in toluene. Geometry optimization of the excited state (S_1_), potential energy surface and natural transition orbits of PDBP-*a,c*, PDBP-*a,i* and PDBP-*b,i* were generated by TDDFT method, respectively, using the same method with S_0_. Huang-Rhys (HR) factors for S_1_ → S_0_ transition were conducted by the MOMAP (Molecular Materials Property Prediction Package)^85,86^. NICS calculations were carried out using the GIAO method^87^, and the Multiwfn^88^ program was used to confirm the coordinates of the ring center. The NICS(1)zz with the ZZ tensor component at the point of 1 Å above the ring center and the NTO analysis was analyzed by Multiwfn^88^. Geometry ESP, geometry comparisons and RMSD values between S_0_ and S_1_ structures were performed by VMD 1.9.3. The Hirshfeld surface^89^ defined by (*d*_i_, *d*_e_) pairs, and the energy frameworks are analyzed by Multiwfn^88^ and VMD 1.9.3. PES for the first excited state of PDBP-*a,i* and PDBP-*b,i* were computed and scanned along the bending angle, which was defined between plane 1 (C1, N1 and N2) and plane 2 (N1, N2 and C2) as a constraint (Supplementary Fig. S11). We progressively scanned the bending angle spanning from 130˚ to 180˚ for every 3˚for PDBP-*a,i* and 2˚ for PDBP-*b,i*.

### Single crystal growth and characterization

Crystals of PDBP-*a,i* and PDBP-*b,i* were grown in tetrahydrofuran/hexane (v:v = 2:1) solution with concentration of 2 mg/mL. Diffraction data of PDBP-*a,i* and PDBP-*b,i* crystals were collected on a Rigaku XtaLAB diffractometer equipped with microfocus sealed X-ray tube (Cu K*α*, *λ* = 1.54184 Å) and hybrid pixel array detector at 150 K. The structures were solved with the direct methods and refined with a full-matrix least-squares technique based on *F*^2^ with the Olex 2 program package. All hydrogen atoms were generated geometrically. [CCDC 2444441 and CCDC 2444438 contains the supplementary crystallographic data for this paper (Supplementary Tables S8−S12). These data can be obtained free of charge from The Cambridge Crystallographic Data Centre via www.ccdc.cam.ac.uk/data_request/cif.]

### Devices preparation

The electroluminescence (EL) devices were fabricated by the vacuum-deposition method. The organic layer passes through a high-vacuum (5 × 10^−6^ Torr) onto a glass substrate pre-coated with an indium tin oxide (ITO) layer with a sheet resistance of 25 Ω square^−1^. The ITO substrates need to be soaked in ultrasonic bath of acetone, isopropanol, detergent, and deionized water respectively for 10 min and dried before it can be transferred to the evaporation chamber for making further efforts. The vacuum-deposited OLEDs were fabricated in the Fangsheng OMV-FS380 vacuum deposition system under a pressure below 5 × 10^−4^ Pa. The organic films, LiF and aluminum were deposited according to the OLED configurations at deposition rates of 1–2 Å s^−1^, 0.1 Å s^−1^ and 3–5 Å s^−1^, respectively. The active area of each device was 3 mm × 3 mm.

The luminance-voltage-current density and external quantum efficiency were characterized with a dual-channel Keithley 2614B source meter and a PIN-25D silicon photodiode. The EL spectra were obtained via an Ocean Optics USB 2000+ spectrometer, along with a Keithley 2614B source meter. The external quantum efficiencies were estimated utilizing the normalized EL spectra and the current efficiencies of the devices.

## Supplementary information

Supplementary information
