# Supplementary material for: Molecular conformation engineering in central 8π-electron system toward unique aggregation-induced ultra-narrowband emission with a FWHM of 13 nm
Source: Light Sci Appl. 2026 Jun 10;15:272. doi: 10.1038/s41377-026-02277-7 (PMC13254055; doi:10.1038/s41377-026-02277-7)
Supplement: Supplementary file 1 — Supplementary information [file 41377_2026_2277_MOESM1_ESM.docx]

Supplementary Materials for

**Molecular conformation engineering in central 8*π*-electron system toward unique aggregation-induced ultra-narrowband emission with a FWHM of 13 nm**

Lu Liu,^1,+^ Han Zhang,^1,2,+,^* Chenfa Xiao,^1,+^ Bingzhu Ma,^1^ Baoxi Li,^1^ Xin He,^4^ Guanjun Xiao,^4^ Bo Zou,^4^ Baolei Tang,^5^ Hongyu Zhang,^5^ Jacky W. Y. Lam,^2^ Ben Zhong Tang,^3,^* and Zhiming Wang^1,^*

*^+^ These authors contribute to this work equally.*

** Corresponding author.*

*E-mail addresses:* [*wangzhiming@scut.edu.cn*](mailto:wangzhiming@scut.edu.cn)*;* [*cheungham@ust.hk*](mailto:cheungham@ust.hk)*; tangbenz@cuhk.edu.cn.*

[**Scheme S1**. Synthetic routes of PDBP*-a*,*c*, PDBP-*a*,*i* and PDBP-*b*,*i*. 4](#_Toc221734239)

[**Figure S1**. ^1^H NMR spectrum of PDBP-*a,c* in CDCl_3_. 5](#_Toc221734240)

[**Figure S2**. ^1^H NMR spectrum of PDBP-*a,i* in *d*_8_*-*THF. 6](#_Toc221734241)

[**Figure S3**. ^13^C NMR spectrum of PDBP-*a,i* in *d*_8_*-*THF. 7](#_Toc221734242)

[**Figure S4**. ^1^H NMR spectrum of PDBP-*b,i* in *d*_8_*-*THF. 8](#_Toc221734243)

[**Figure S5**. ^13^C NMR spectrum of PDBP-*b,i* in *d*_8_*-*THF. 9](#_Toc221734244)

[**Table S1**. Photopysics properties of PDBP-*a,c*, PDBP-*a,i*, and PDBP-*b,i* in toluene (10^−5^ M). 9](#_Toc221734245)

[**Figure S6**. Photoluminescence spectra of PDBP-*a,i* and PDBP-*b,i* in different solvent (10^−5^ M). 10](#_Toc221734246)

[**Figure S7**. Reconstructed transient fluorescence of (A) PBDP-*b*,*i* and (C) PBDP-*b*,*i* at different times in a toluene solution with concentration of 10^−5^ M at room temperature by TCSPC. Fluorescence spectra decay traces recorded for (B) PBDP-*b*,*i* and (D) PBDP-*b*,*i* in a toluene solution with concentration of 10^−5^ M at room temperature. 10](#_Toc221734247)

[**Table S2**. Maximun emission peak of PDBP-*a,i* and PDBP-*b,i* in different solvent (10^−5^ M). 11](#_Toc221734248)

[**Figure S8**. Optimized geometry of *ax-ax* conformation PDBP-*a,c* at S_0_. 11](#_Toc221734249)

[**Figure S9**. Optimized geometry of *ax-eq* conformation PDBP-*a,i* in toluene at S_0_. 11](#_Toc221734250)

[**Figure S10.** Optimized geometry of *eq-eq* conformation PDBP-*b,i* in toluene at S_0_. 12](#_Toc221734251)

[**Figure S11**. Calculated PES scanned along the change of *α* in dihydrophenazine center at the S_1_ state for (A) PDBP-*a,i* and (B) PDBP-*b,i*. 12](#_Toc221734252)

[**Figure S12**. NICS(1)_ZZ values of the R, R*, I, I* and P, P* states for (a) PDBP-*a,c*, (b) PDBP-*a,i* and (c) PDBP-*b,i*. Calculated at B3LYP/6-311G(d,p) level. Note: R/R* represent the S_0_ geometry corresponding to the global minimum, I/I* and P/P* represent the S_1_ geometry at different local minimum. R, I and P are calculated in the ground state. R*, I* and P* represent the vertical transitions from R, I and P, respectively, which are calculated at the T_1_ states as a representative case in the excited state. 13](#_Toc221734253)

[**Figure S13**. Optimized geometry of *eq-eq* conformation PDBP-*a,c* at S_1_. 13](#_Toc221734254)

[**Figure S14**. Optimized geometry of *ax-eq* conformation PDBP-*a,i* at S_1_. 14](#_Toc221734255)

[**Figure S15**. Optimized geometry of *eq-eq* conformation PDBP-*a,i* at S_1_. 14](#_Toc221734256)

[**Figure S16**. Optimized geometry of *eq-eq* conformation PDBP-*b,i* at S_1_. 14](#_Toc221734257)

[**Table S3**. The calculated energy levels, oscillator strengths (*f*) and orbital transition analyses for PDBP-*a,c*, PDBP-*a,i*, and PDBP-*b,i*. 15](#_Toc221734258)

[**Figure S17**. The RMSD values between the S_0_ and S_1_ states for PDBP-*a,c* (A), PDBP-*a,i* (B, C) and PDBP-*b,i* (D). 15](#_Toc221734259)

[**Figure S18**. Simulated vibrationally-resolved electronic spectra of PDBP-*b,i*. 16](#_Toc221734260)

[**Figure S19**. PL spectra of PDBP-*a,i* (A) in solid powders. Structural information for PDBP-*a,i* (B) in crystal (Left: Packing structures; Right: Intermolecular interaction. H-atoms were omitted for clear). 16](#_Toc221734261)

[**Figure S20**. Transient PL decay curves of (A) PDBPP-*a,i* and (B) PDBP-*b,i* in solid powders at *λ*_ex_ = 340 nm. 16](#_Toc221734262)

[**Table S4**. Photopysics properties of PDBP-*a,i* and PDBP-*b,i* in solid powders. 16](#_Toc221734263)

[**Figure S21**. Monomer structure for PBDP-*a,i* in crystal. 17](#_Toc221734264)

[**Figure S22**. Packing structure of PDBP-*a,i* in crystal. 17](#_Toc221734265)

[**Figure S23**. Monomer structure for PBDP-*b,i* in crystal. 17](#_Toc221734266)

[**Figure S24**. Hirshfeld surface for one PDBP-*b,i* mapped from red (distance shorter than sum of Van der Waals radii) through white to blue (distances longer than sum of Van der Waals radii). The 2D histogram is a function of the number of surface points. 18](#_Toc221734267)

[**Pressure-dependent PL measurements** 18](#_Toc221734268)

[**Figure S25**. PL spectra evolution of PDBP-*b,i* in solid powders with increasing pressure from atmospheric to 7.3 GPa. 19](#_Toc221734269)

[**Figure S26**. (A) TGA analysis and (B) DSC curve of PDBP-*b,i* in solid powders. 19](#_Toc221734270)

[**Figure S27**. Cyclic voltammograms of PDBP-*b,i* measured in DCM containing 0.1 M tetra-*n*-butylammonium hexafluorophosphate. 20](#_Toc221734271)

[**Figure S28.** Device structure, HOMO and LUMO for each material. 20](#_Toc221734272)

[**Figure S29**. PL spectra for PBDP-*b,i* in doped film (in mCBP). The spectral narrowing mechanism of PDBP-*b,i* in host mCBP originated from aggregation-induced restriction of high-frequency vibration. 21](#_Toc221734273)

[**Figure S30.** Low-temperature phospherescence spectrum in frozen toluene matrix (10^−5^ M) for PDBP-*b,i*. 21](#_Toc221734274)

[**Table S5**. OLED performace of PDBP-*b*,*i*. 21](#_Toc221734275)

[**Singlet-singlet annihilation (SSA),^1^ singlet-heat annihilation (SHA)^1^ and singlet-polaron annihilation (SPA)^2^ fitting methods of PDBP-*b,i* based OLEDs.** 22](#_Toc221734276)

[**Figure S31.** *η*_ext_−*J* characteristics of PDBP-*b,i* devices at doping concentration of 30 wt% with SSA, SHA and SPA models. Best fit was obtained with $J0$ = 18.85 mA/cm^2^, *m =* 0.7, $\tau2ksqkth$ = 1$\times$10^−10^, *l* = 1.5. 23](#_Toc221734277)

[**Table S6**. Summary of the reported doped OLEDs with FWHM ≤ 25 nm. 23](#_Toc221734278)

[**Table S7.** Summary of the reported high doping concentration (≥ 10 wt%) OLEDs with FWHM ≤ 40 nm. 29](#_Toc221734279)

[**Table S8**. Crystal data and structure refinements for PDBP-*a*,*i* and PDBP-*b*,*i*. 33](#_Toc221734280)

[**Table S9**. Bond lengths for PDBP-*a,i*. 35](#_Toc221734281)

[**Table S10**. Bond angles for PDBP-*a,i*. 36](#_Toc221734282)

[**Table S11**. Bond lengths for PDBP-*b,i*. 37](#_Toc221734283)

[**Table S12**. Bond angles for PDBP-*b,i.* 38](#_Toc221734284)

[**References** 39](#_Toc221734285)

**
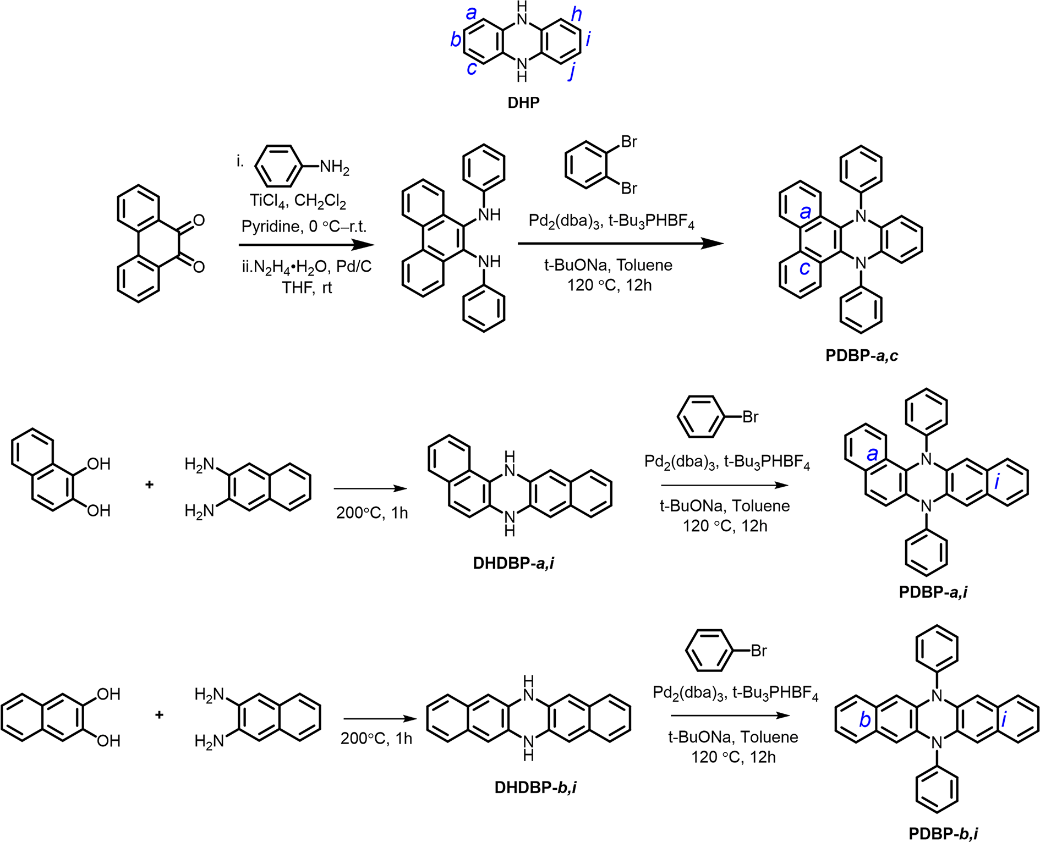
**

**Scheme S1**. Synthetic routes of PDBP*-a*,*c*, PDBP-*a*,*i* and PDBP-*b*,*i*.

**9,14-diphenyl-9,14-dihydrodibenzo[*a*,*c*]phenazine (PDBP*-a*,*c*)**: HRMS (ESI): C_32_H_23_N_2_^+^ ([M+H]^+^), calcd. m/z = 435.1856, found m/z = 435.1861. ^1^H NMR (500 MHz, CDCl_3_) *δ* 8.74 (d, *J* = 8.3 Hz, 2H), 8.12 (d, *J* = 8.1 Hz, 2H), 7.75 (dd, *J* = 5.9, 3.5 Hz, 2H), 7.64 (t, *J* = 7.6 Hz, 2H), 7.54 (t, *J* = 7.6 Hz, 2H), 7.34 (dt, *J* = 5.9, 3.7 Hz, 2H), 7.01 (dd, *J* = 8.7, 6.9 Hz, 4H), 6.97 (d, *J* = 8.1 Hz, 4H), 6.78 (t, *J* = 7.1 Hz, 2H).

**9,14-diphenyl-9,14-dihydrodibenzo[*a*,*i*]phenazine (****PDBP*-a*,*i*)**:

HRMS (ESI): C_32_H_22_N_2_^+^ ([M]^+^), calcd. m/z = 434.1783, found m/z = 434.1790. ^1^H NMR (400 MHz, THF) *δ* 8.10 (d, *J* = 7.5 Hz, 2H), 7.88 – 7.81 (m, 1H), 7.82 – 7.76 (m, 1H), 7.73 (t, *J* = 7.7 Hz, 2H), 7.68 – 7.62 (m, 1H), 7.61 – 7.55 (m, 1H), 7.52 – 7.46 (m, 3H), 7.40 (ddd, *J* = 8.3, 6.8, 1.3 Hz, 1H), 7.34 – 7.23 (m, 3H), 7.14 – 7.07 (m, 4H), 6.89 (tt, *J* = 6.2, 2.1 Hz, 1H), 6.78 (d, *J* = 9.0 Hz, 1H), 6.68 (s, 1H). ^13^C NMR (101 MHz, THF) *δ* 150.55, 142.89, 140.05, 139.81, 134.69, 132.24, 130.90, 130.75, 130.13, 130.03, 129.96, 128.62, 128.55, 127.85, 127.01, 126.34, 126.26, 125.66, 125.54, 125.03, 124.07, 123.40, 123.29, 122.45, 121.70, 118.81, 115.90, 109.47, 67.01, 66.88, 66.79, 66.66, 66.57, 66.44, 66.22, 66.00, 24.88, 24.75, 24.68, 24.65, 24.55, 24.48, 24.45, 24.35, 24.15, 23.96, 22.59, 13.46.

**9,14-diphenyl-9,14-dihydrodibenzo[*b*,*i*]phenazine (PDBP*-b*,*i*):**

HRMS (ESI): C_32_H_22_N_2_^+^ ([M]^+^), calcd. m/z = 434.1783, found m/z = 434.1787. ^1^H NMR (400 MHz, THF) *δ* 7.80 (t, *J* = 7.8 Hz, 1H), 7.70 – 7.61 (m, 1H), 7.56 – 7.50 (m, 1H), 7.10 – 6.88 (m, 2H), 5.98 (s, 1H). ^13^C NMR (126 MHz, THF) δ 139.95, 135.09, 131.50, 130.75, 130.26, 128.69, 125.43, 123.41, 107.76.

**Figure S1**. ^1^H NMR spectrum of PDBP-*a,c* in CDCl_3_.

**Figure S2**. ^1^H NMR spectrum of PDBP-*a,i* in *d*_8_*-*THF.

**Figure S3**. ^13^C NMR spectrum of PDBP-*a,i* in *d*_8_*-*THF.

**Figure S4**. ^1^H NMR spectrum of PDBP-*b,i* in *d*_8_*-*THF.

**Figure S5**. ^13^C NMR spectrum of PDBP-*b,i* in *d*_8_*-*THF.

**Table S1**. Photopysics properties of PDBP-*a,c*, PDBP-*a,i*, and PDBP-*b,i* in toluene (10^−5^ M).

|  | ***λ*_abs_**  **(nm)** | ***ε***  **(mol×L^−1^×cm^−1^)** | ***λ*_em_**  **(nm)** | ***η*_PL_**  **(%)** | ***k*_r_ (s^−1^)** | ***k*_nr_ (s^−1^)** |
| --- | --- | --- | --- | --- | --- | --- |
| **PDBP-*a,c*** | 350 | 7447 | 595 | 22 | 3.17$\times$10^7^ | 1.13$\times$10^8^ |
| **PDBP-*a,i*** | 418 | 1569 | 425 | 48 | 1.78$\times$10^8^ | 1.93$\times$10^8^ |
|  |  |  | 500 |  | 5.33$\times$10^7^ | 5.78$\times$10^7^ |
| **PDBP-*b,i*** | 418 | 39640 | 425 | 75 | 2.63$\times$10^8^ | 8.77$\times$10^7^ |

**
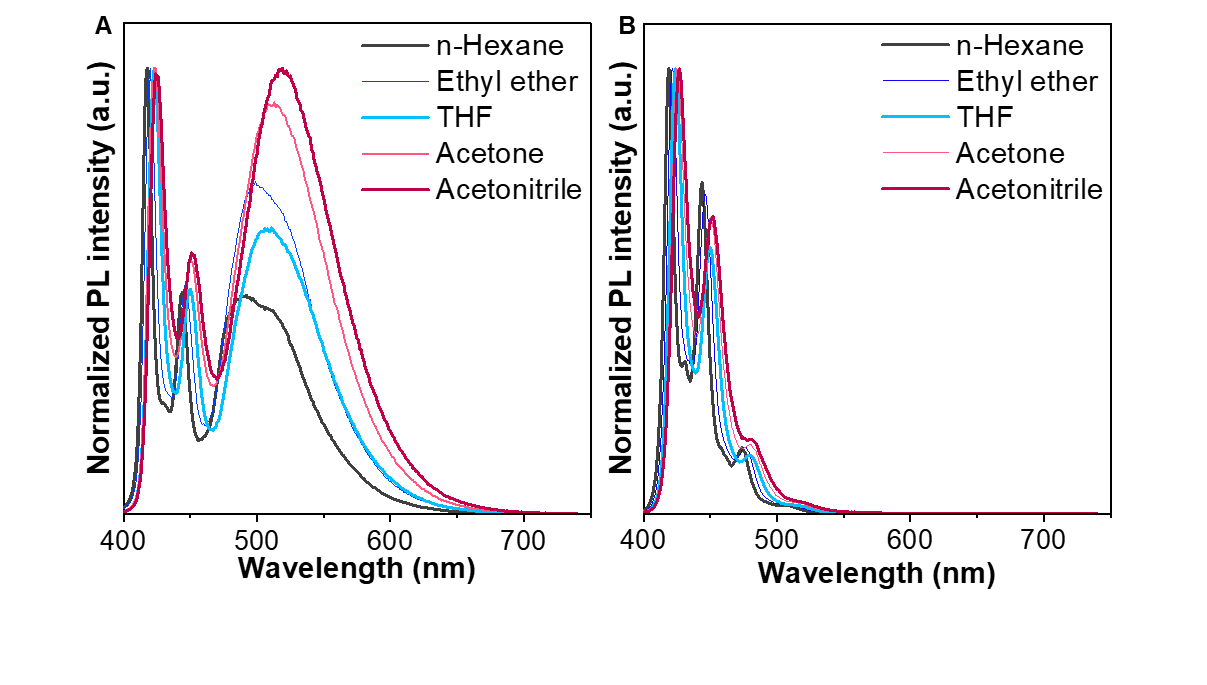
**

**Figure S6**. Photoluminescence spectra of (A) PDBP-*a,i* and (B) PDBP-*b,i* in different solvent (10^−5^ M).


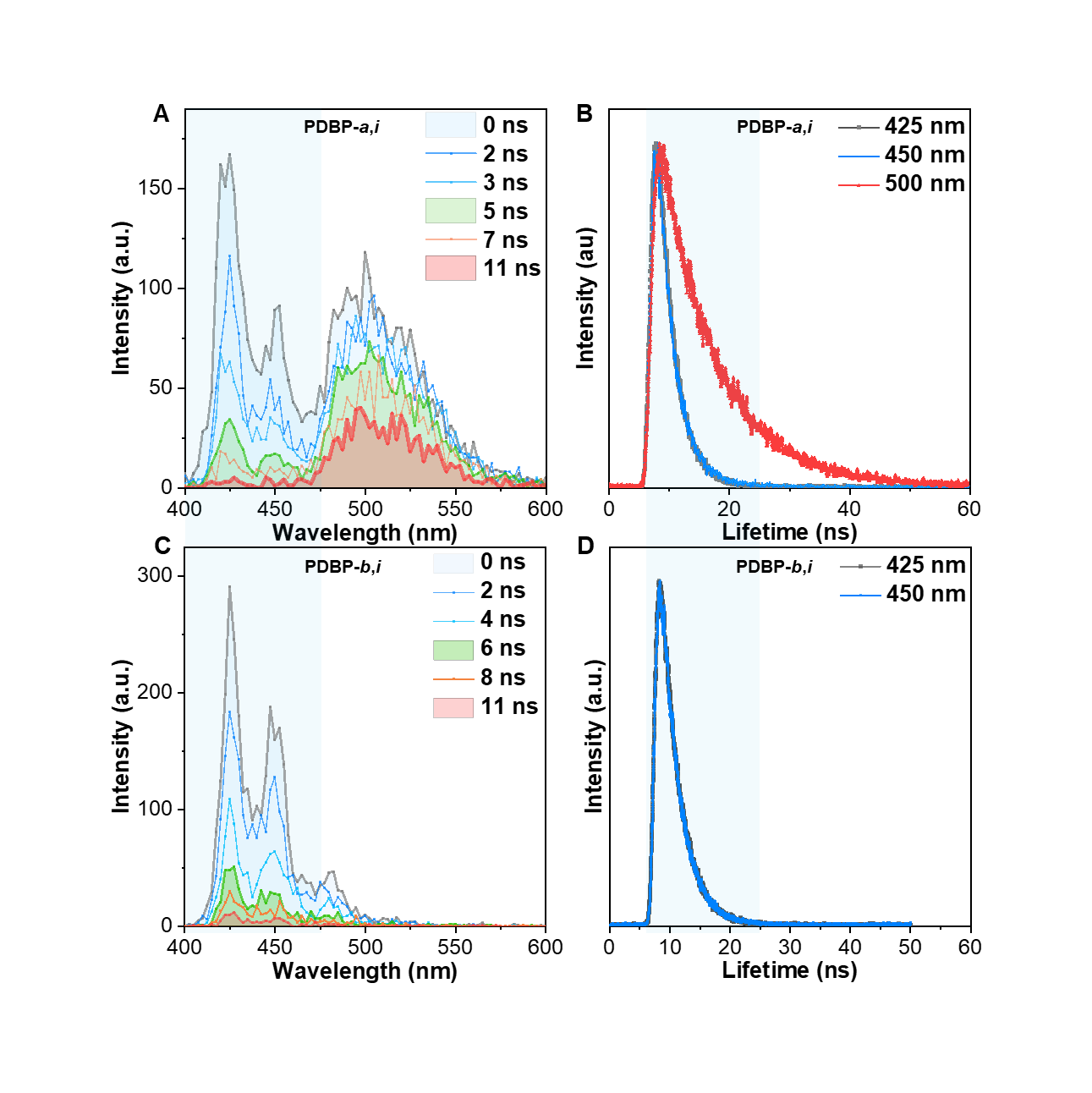


**Figure S7**. Reconstructed transient fluorescence of (A) PBDP-*b*,*i* and (C) PBDP-*b*,*i* at different times in a toluene solution with concentration of 10^−5^ M at room temperature by TCSPC. Fluorescence spectra decay traces recorded for (B) PBDP-*b*,*i* and (D) PBDP-*b*,*i* in a toluene solution with concentration of 10^−5^ M at room temperature.

**Table S2**. Maximun emission peak of PDBP-*a,i* and PDBP-*b,i* in different solvent (10^−5^ M).

|  | ***n-*Hexane** | **Ethyl ether** | **THF** | **Acetone** | **Acetonitrile** |
| --- | --- | --- | --- | --- | --- |
| **PDBP-*a,i*** | 418/490 | 420/499 | 423/507 | 424/511 | 424/518 |
| **PDBP-*b,i*** | 419 | 422 | 424 | 425 | 427 |


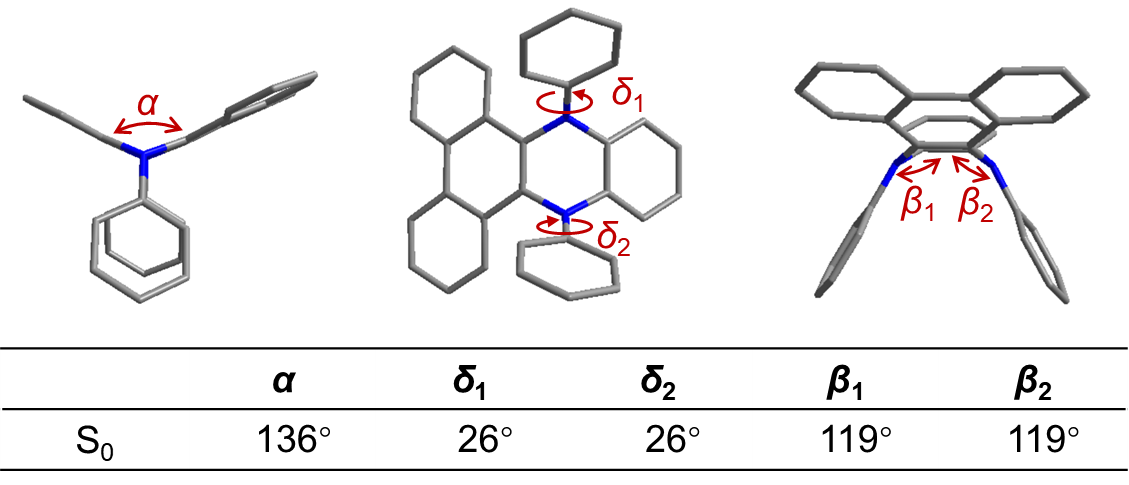


**Figure S8**. Optimized geometry of *ax-ax* conformation PDBP-*a,c* at S_0_.


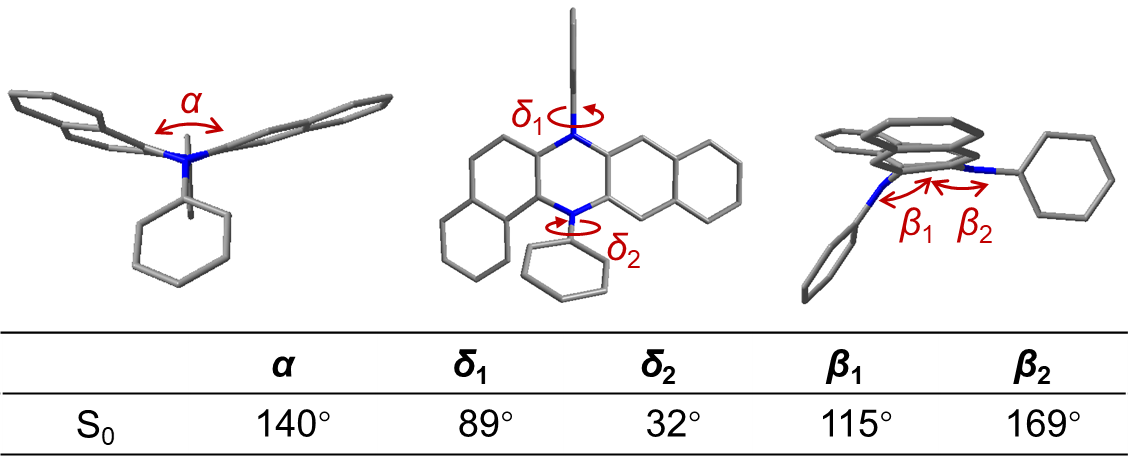


**Figure S9**. Optimized geometry of *ax-eq* conformation PDBP-*a,i* in toluene at S_0_.


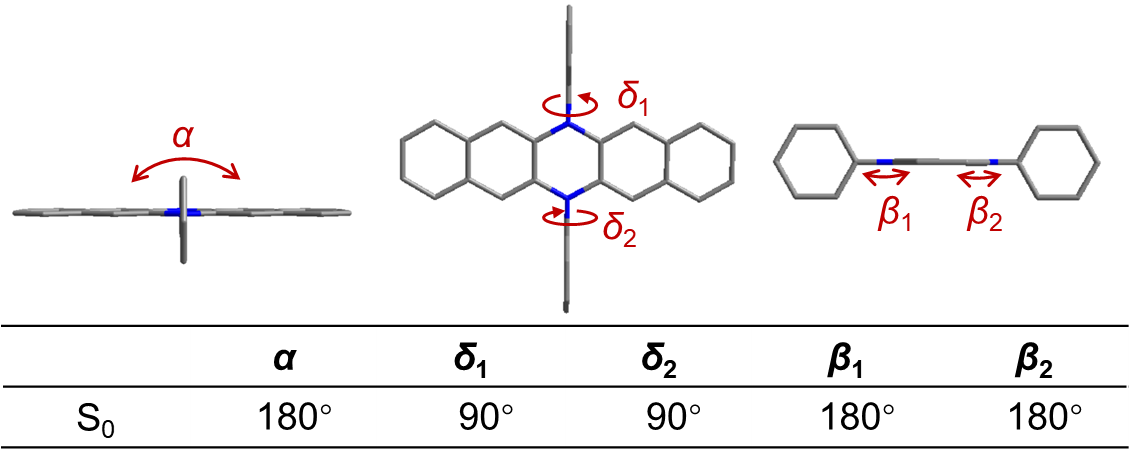


**Figure S10.** Optimized geometry of *eq-eq* conformation PDBP-*b,i* in toluene at S_0_.


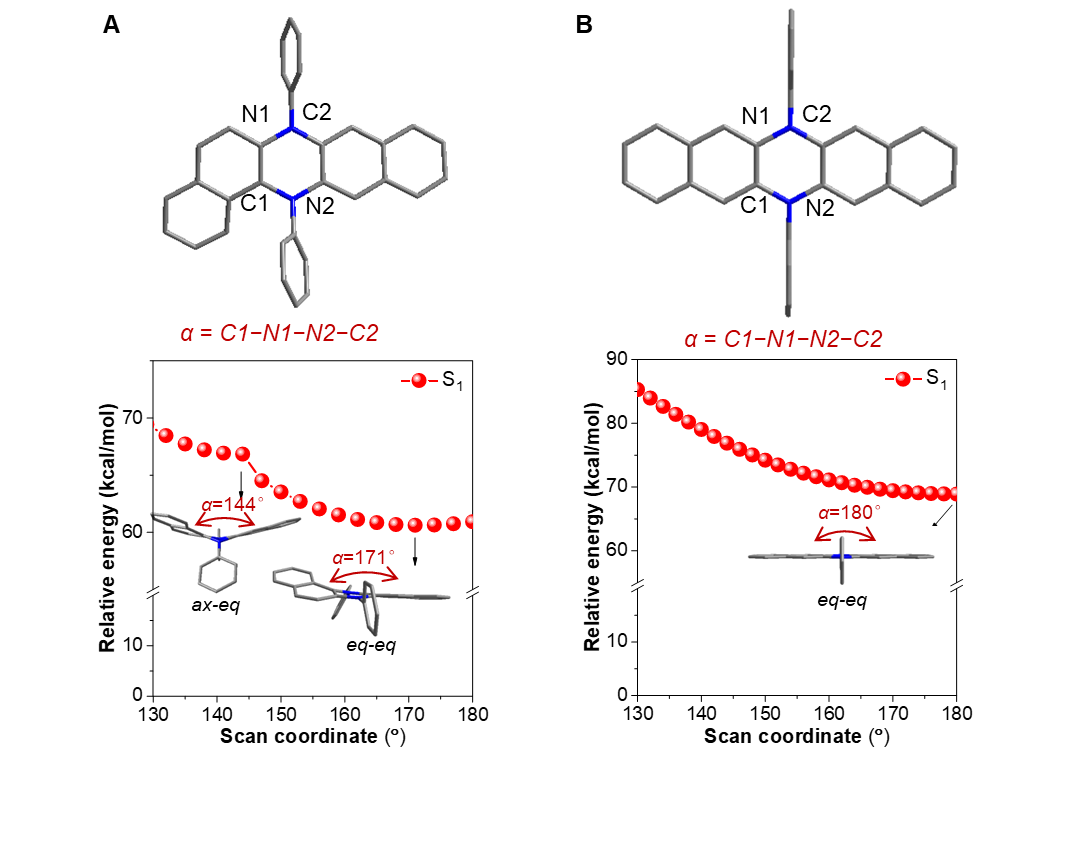


**Figure S11**. Calculated PES scanned along the change of *α* in dihydrophenazine center at the S_1_ state for (A) PDBP-*a,i* and (B) PDBP-*b,i*.


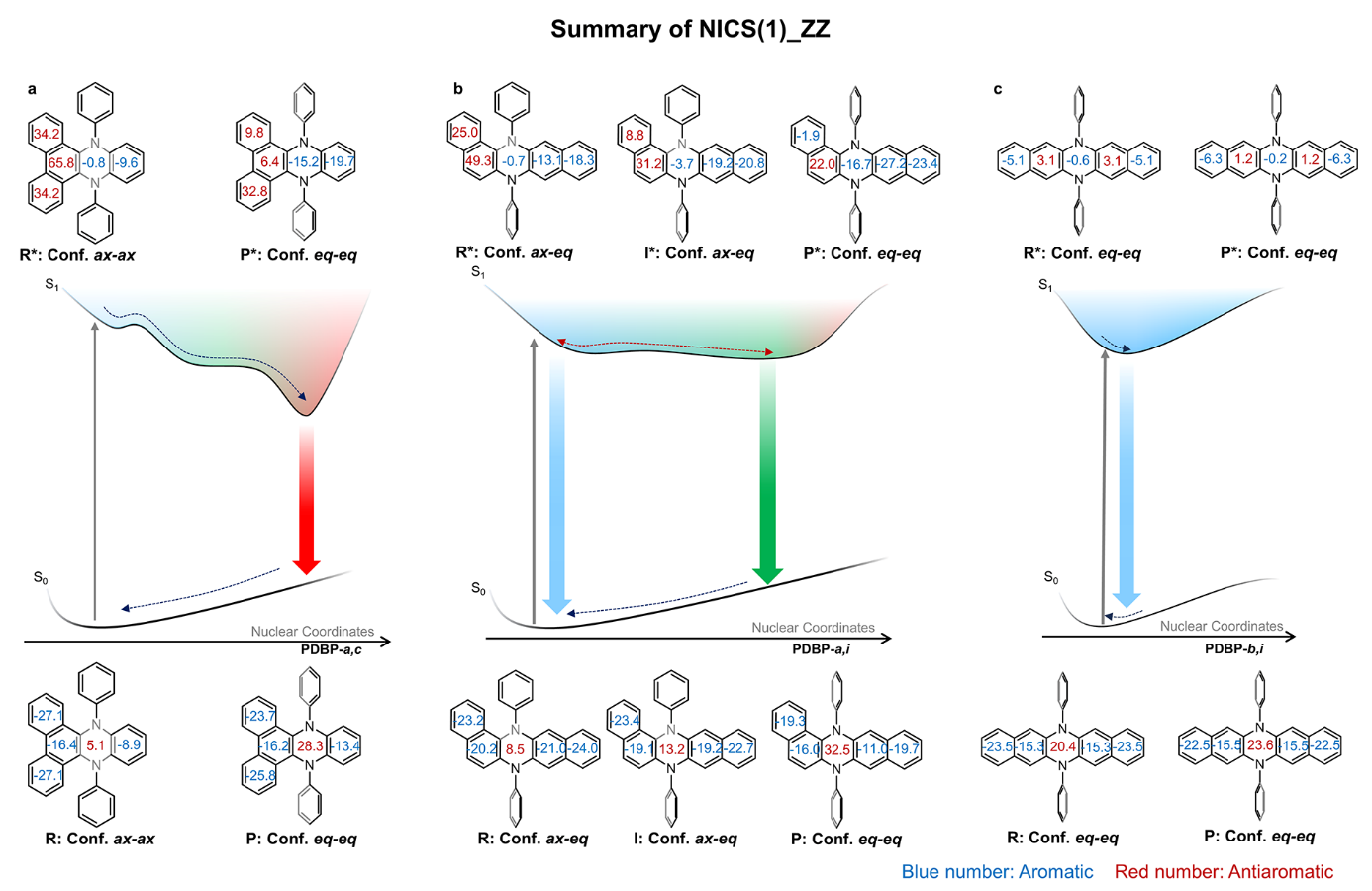


**Figure S12**. NICS(1)_ZZ values of the R, R*, I, I* and P, P* states for (a) PDBP-*a,c*, (b) PDBP-*a,i* and (c) PDBP-*b,i*. Calculated at B3LYP/6-311G(d,p) level. Note: R/R* represent the S_0_ geometry corresponding to the global minimum, I/I* and P/P* represent the S_1_ geometry at different local minimum. R, I and P are calculated in the ground state. R*, I* and P* represent the vertical transitions from R, I and P, respectively, which are calculated at the T_1_ states as a representative case in the excited state.


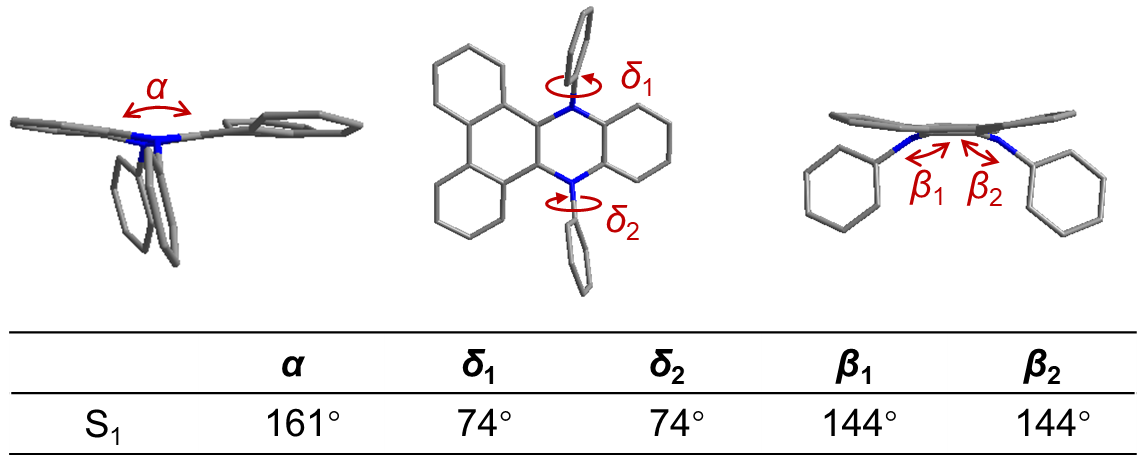


**Figure S13**. Optimized geometry of *eq-eq* conformation PDBP-*a,c* at S_1_.


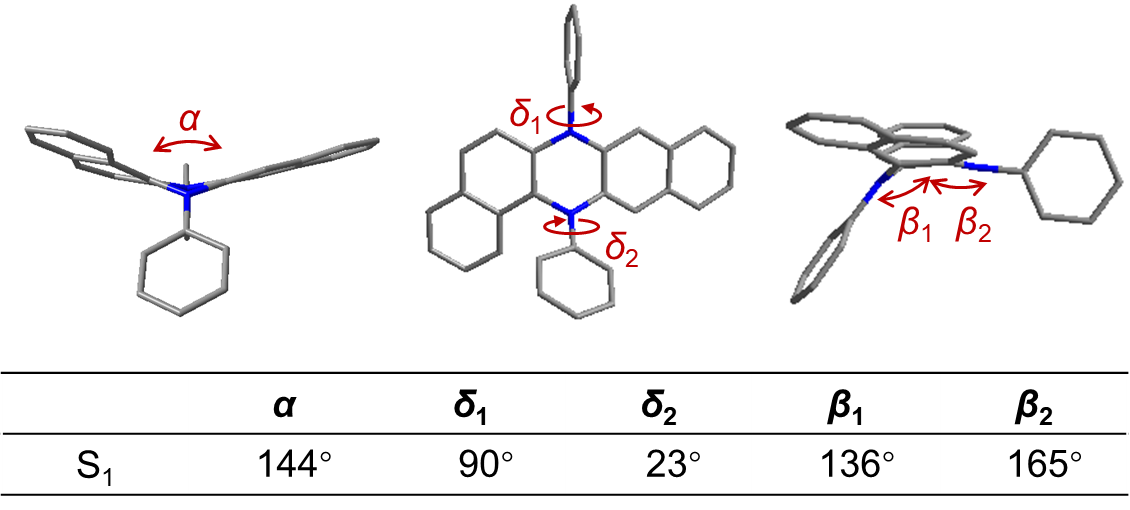


**Figure S14**. Optimized geometry of *ax-eq* conformation PDBP-*a,i* at S_1_.


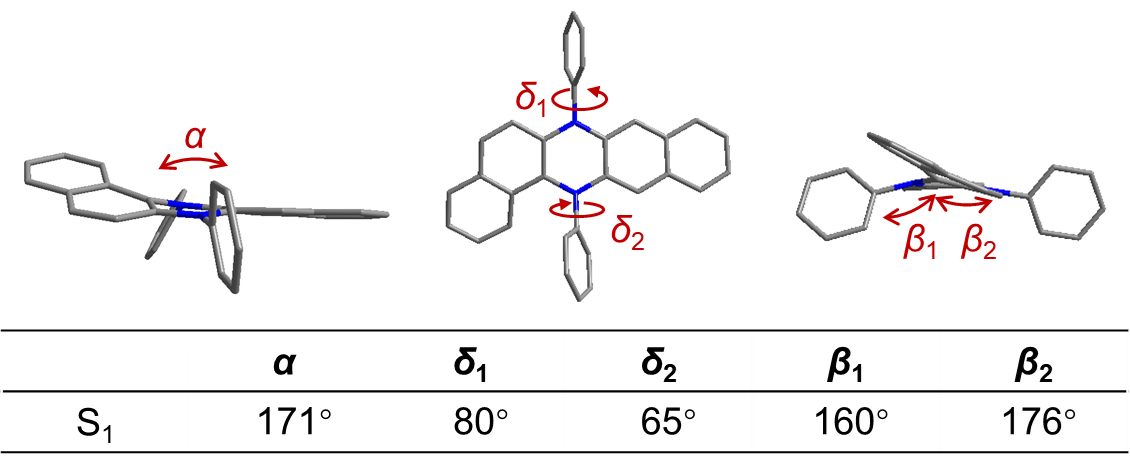


**Figure S15**. Optimized geometry of *eq-eq* conformation PDBP-*a,i* at S_1_.


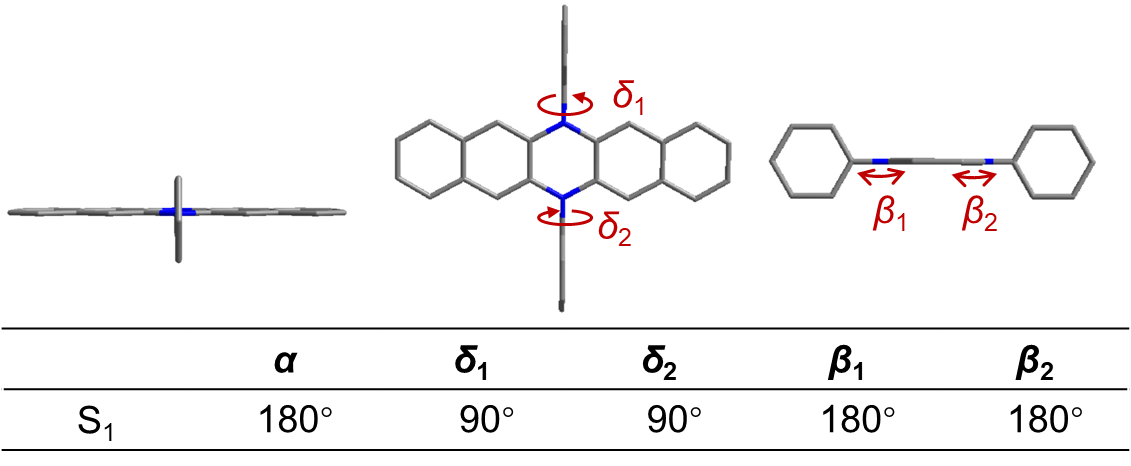


**Figure S16**. Optimized geometry of *eq-eq* conformation PDBP-*b,i* at S_1_.

**Table S3**. The calculated energy levels, oscillator strengths (*f*) and orbital transition analyses for PDBP-*a,c*, PDBP-*a,i*, and PDBP-*b,i*.

|  | **Absorption S_0_→S_1_** | | **Emission S_1_→S_0_** | | | **Stokes shift (nm)** | |
| --- | --- | --- | --- | --- | --- | --- | --- |
|  | ***λ*_exp._(nm)** | ***λ*_calc._(nm)-** | ***λ*_exp._(nm)** | ***λ*_calc._(nm)** | ***f*** | **ΔE_exp._** | **ΔE_calc._** |
| **PDBP-*a,c*** | 350 | 385 | 427/595 | 463/561 | 0.09/0.08 | 77/245 | 78/225 |
| **PDBP-*a,i*** | 418 | 388 | 425/500 | 473/561 | 0.05/0.03 | 7/82 | 85/173 |
| **PDBP-*b,i*** | 418 | 404 | 425 | 427 | 0.55 | 7 | 23 |


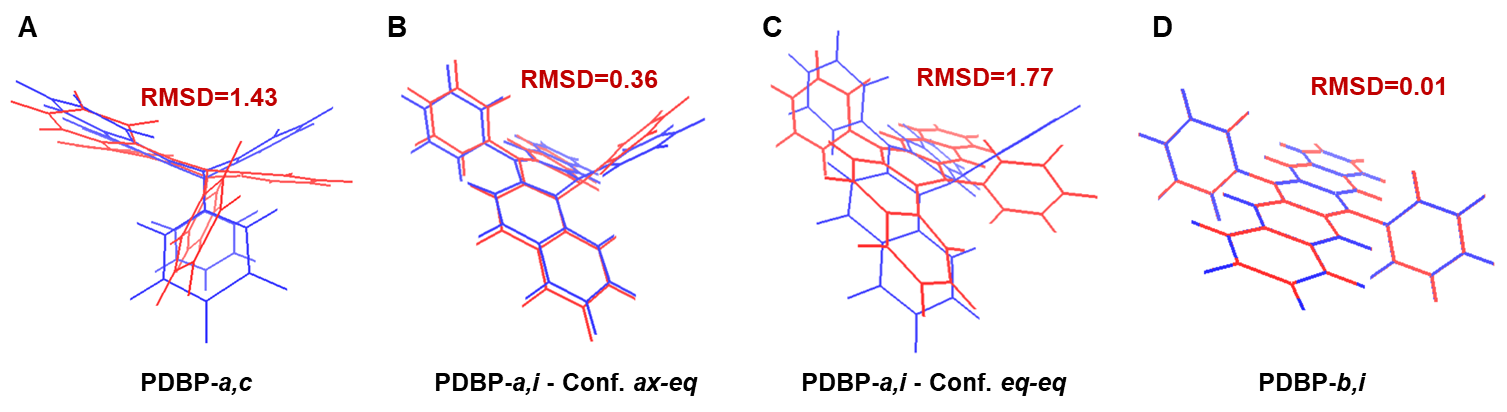


**Figure S17**. The RMSD values between the S_0_ and S_1_ states for PDBP-*a,c* (A), PDBP-*a,i* (B, C) and PDBP-*b,i* (D).


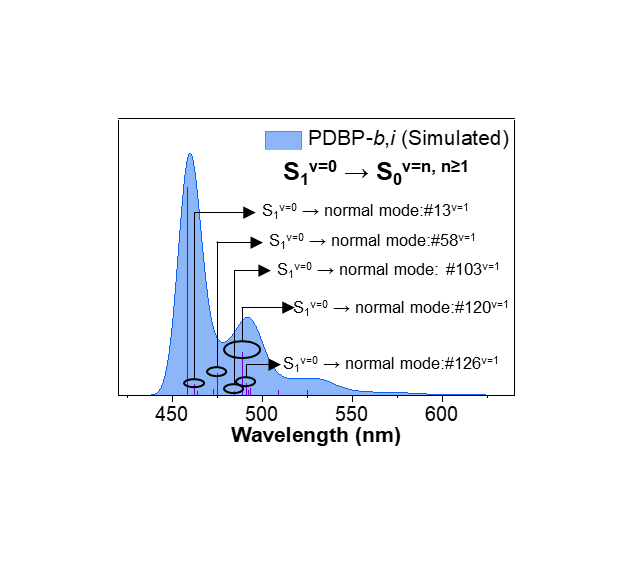


**Figure S18**. Simulated vibrationally-resolved electronic spectra of PDBP-*b,i*.


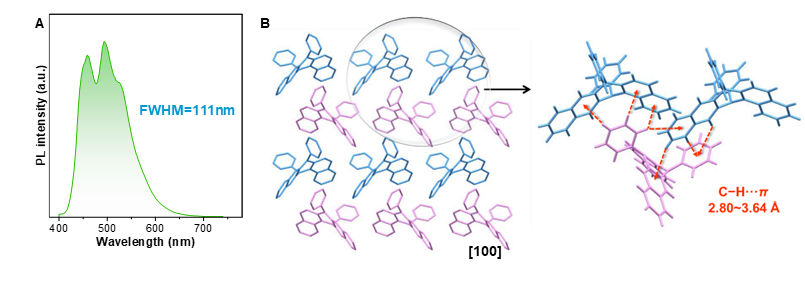


**Figure S19**. PL spectra of (A) PDBP-*a,i* in solid powders. Structural information for (B) PDBP-*a,i* in crystal (Left: Packing structures; Right: Intermolecular interaction. H-atoms were omitted for clear).


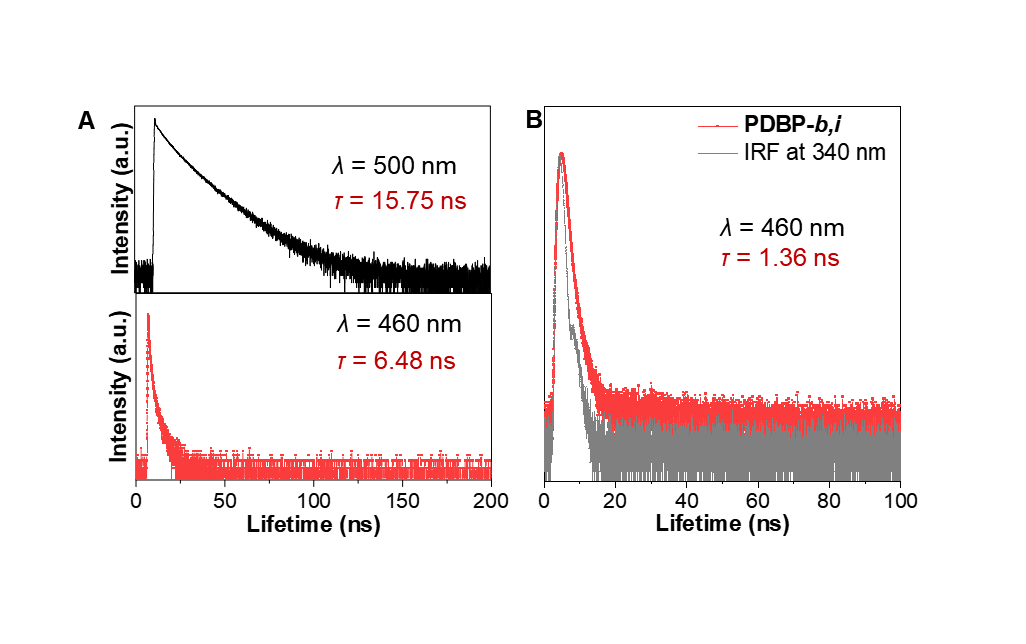


**Figure S20**. Transient PL decay curves of (A) PDBPP-*a,i* and (B) PDBP-*b,i* in solid powders at *λ*_ex_ = 340 nm.

**Table S4**. Photopysics properties of PDBP-*a,i* and PDBP-*b,i* in solid powders.

|  | ***λ*_em_ (nm)** | ***τ*(ns)** | ***η*_PL_ (%)** | ***K*_r_** **(s^−1^)** | ***K*_nr_** **(s^−1^)** |
| --- | --- | --- | --- | --- | --- |
| **PDBP-*a,i*** | 460 | 6.48 | 33 | 5.09$\times$10^7^ | 1.03$\times$10^8^ |
|  | 500 | 15.75 |  | 2.10$\times$10^7^ | 4.25$\times$10^7^ |
| **PDBP-*b,i*** | 460 | 1.36 | 18 | 1.32$\times$10^8^ | 6.03$\times$10^8^ |


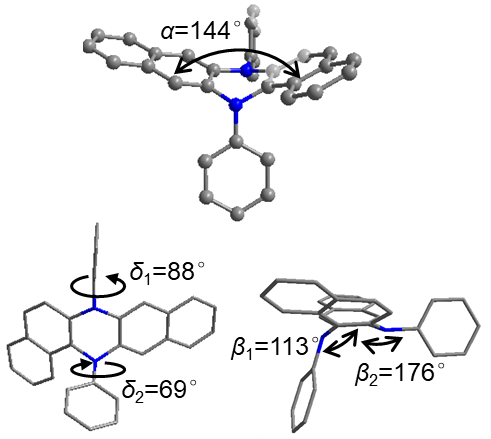


**Figure S21**. Monomer structure for PBDP-*a,i* in crystal.


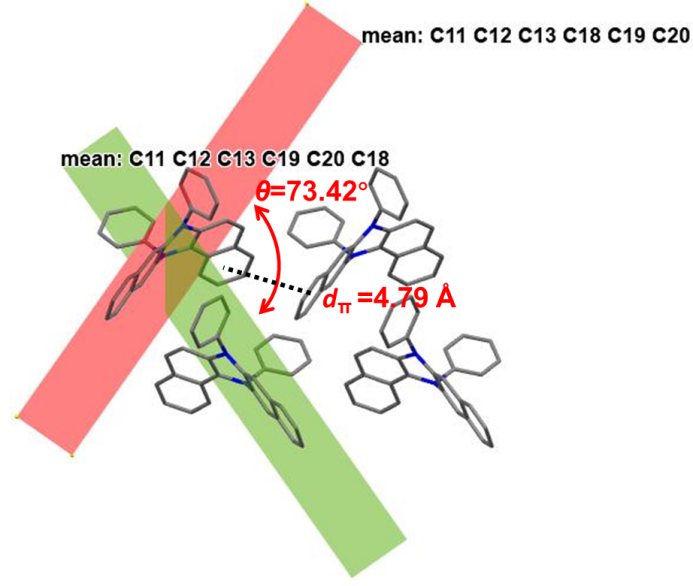


**Figure S22**. Packing structure of PDBP-*a,i* in crystal.


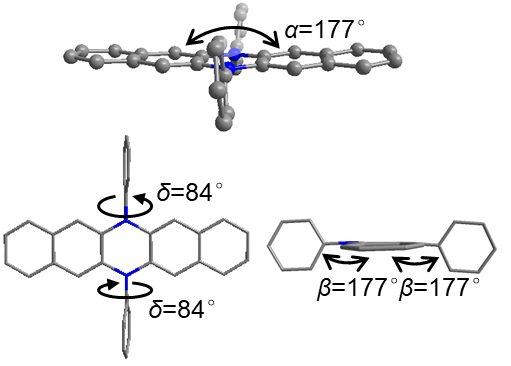


**Figure S23**. Monomer structure for PBDP-*b,i* in crystal.


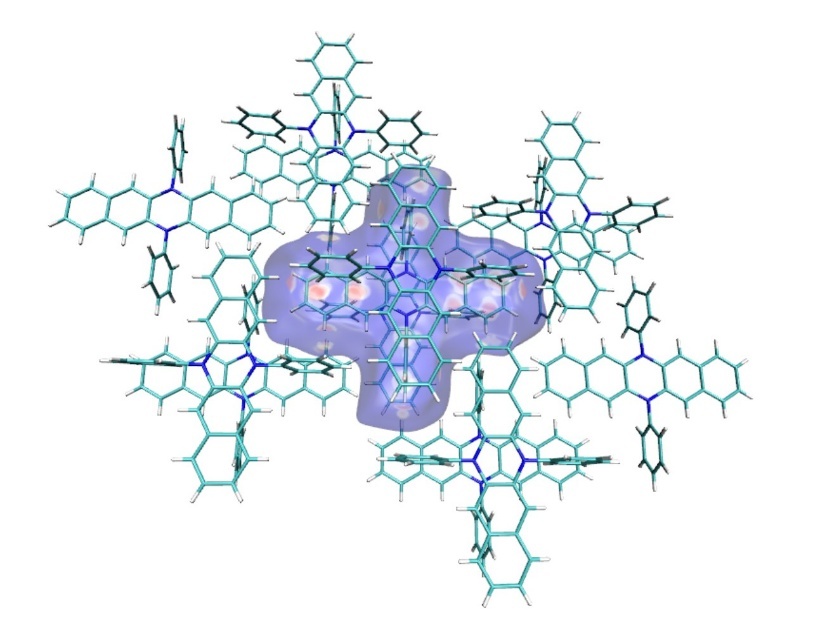


**Figure S24**. Hirshfeld surface for one PDBP-*b,i* mapped from red (distance shorter than sum of Van der Waals radii) through white to blue (distances longer than sum of Van der Waals radii). The 2D histogram is a function of the number of surface points.

**Pressure-dependent PL measurements**

All of high-pressure experiments were carried out with a symmetric diamond anvil cell (DAC). The sample and a small ruby were loaded into 150 *μ*m-diameter chamber of a DAC, constructed from a T301 steel gasket preindented to a thickness of 45 *μ*m. The pressure calibration was determined utilizing the standard ruby fluorescent technique. In high-pressure experiments, silicon oil was utilized as the pressure transmitting medium (PTM) for optical absorption and PL experiments. The PTM did not have any detectable effect on the behavior of sample under high pressure. All of the measurements were performed at room temperature.

Note that during the experiments, all the parameters are fixed completely over each high-pressure PL experiment to avoid the effects of different excitation laser intensities and luminous fluxes on the resulting PL intensity of intrinsic sample. High-pressure PL spectra of sample were recorded with an optical fiber spectrometer (Ocean Optics, QE65000).


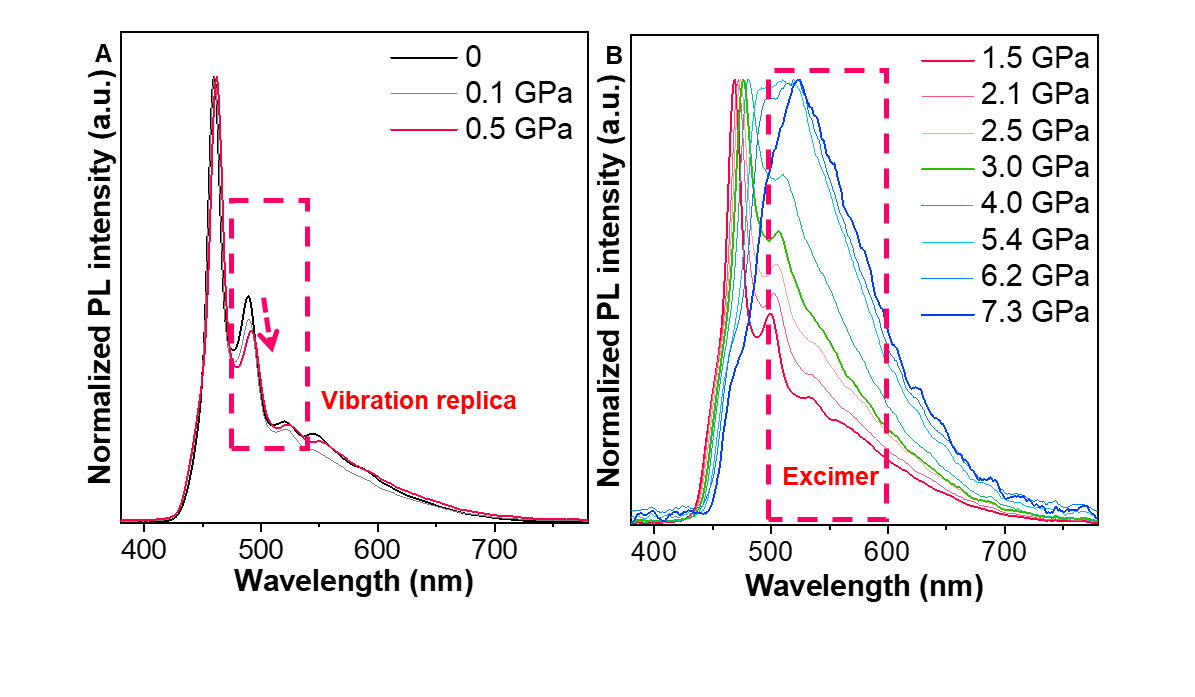


**Figure S25**. PL spectra evolution of PDBP-*b,i* in solid powders with increasing pressure from atmospheric to 7.3 GPa.


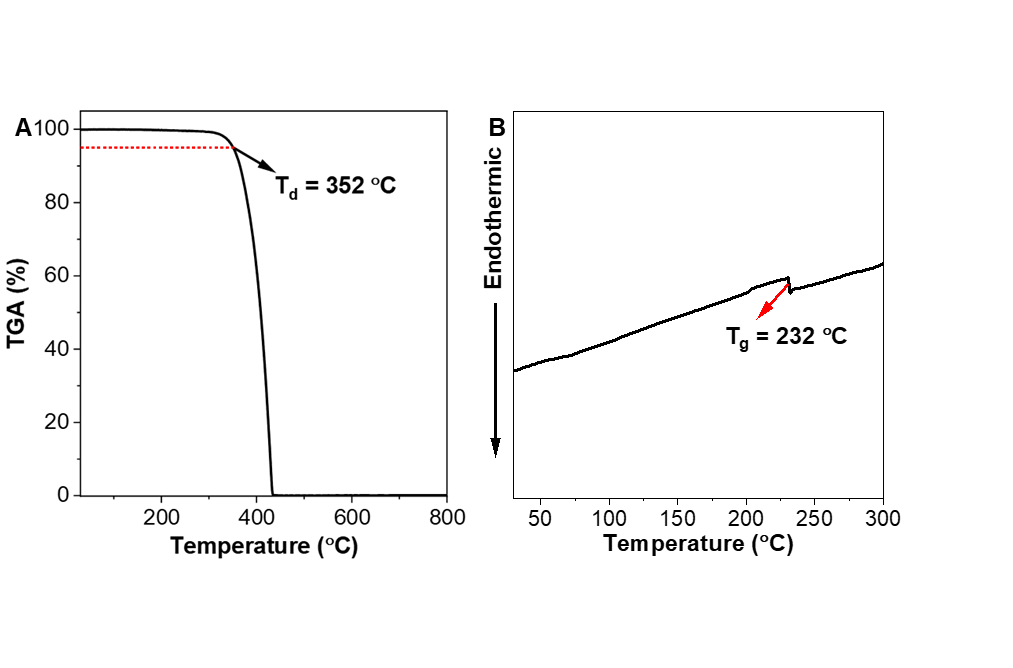


**Figure S26**. (A) TGA analysis and (B) DSC curve of PDBP-*b,i* in solid powders.


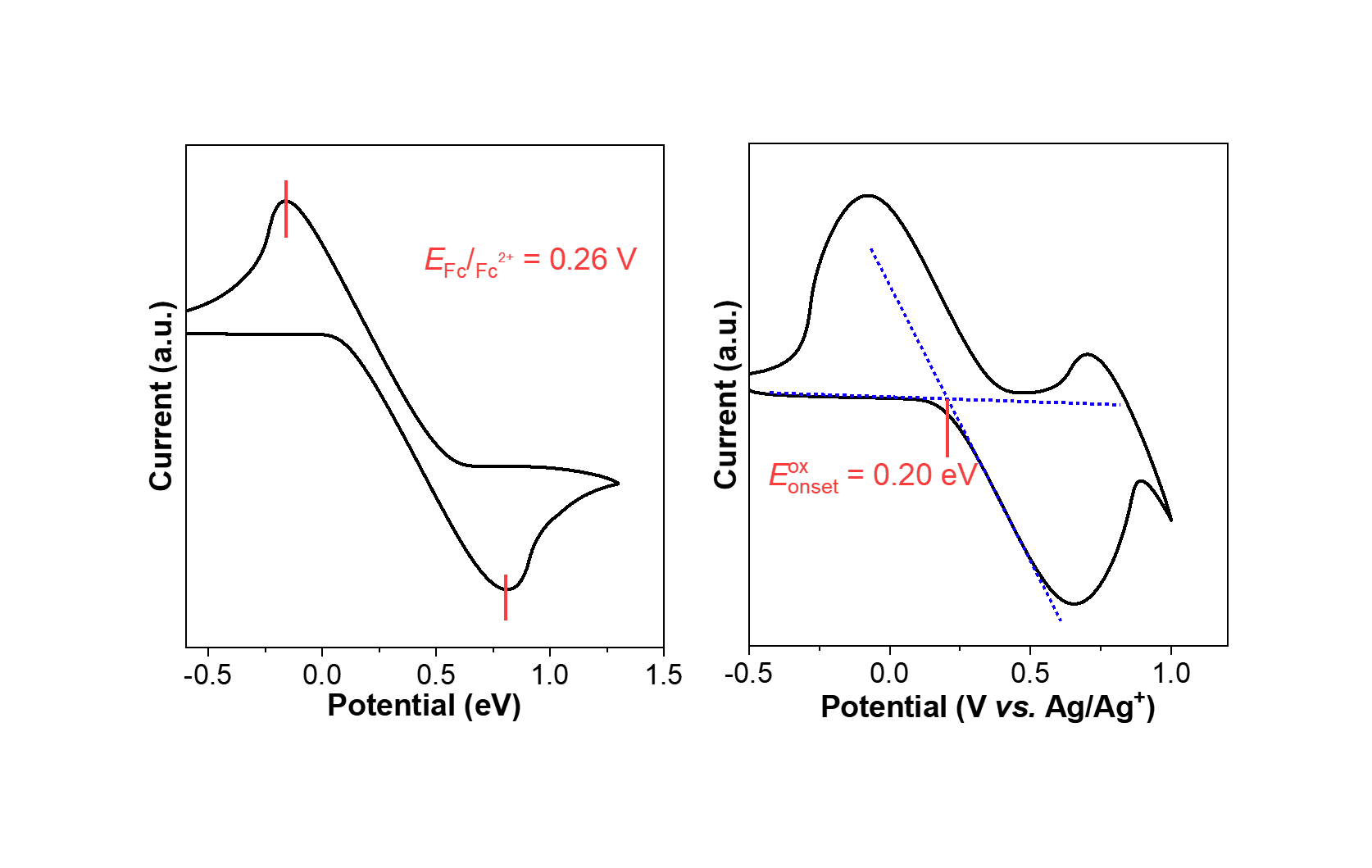


**Figure S27**. Cyclic voltammograms of PDBP-*b,i* measured in DCM containing 0.1 M tetra-*n*-butylammonium hexafluorophosphate.


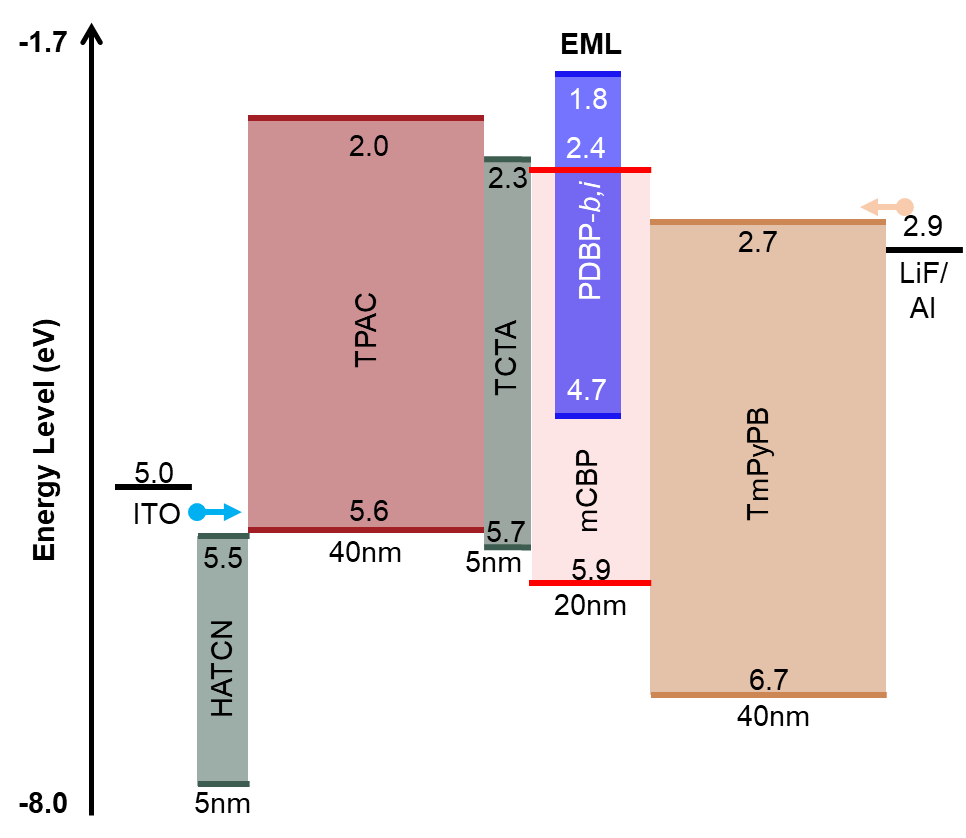


**Figure S28.** Device structure, HOMO and LUMO for each material.


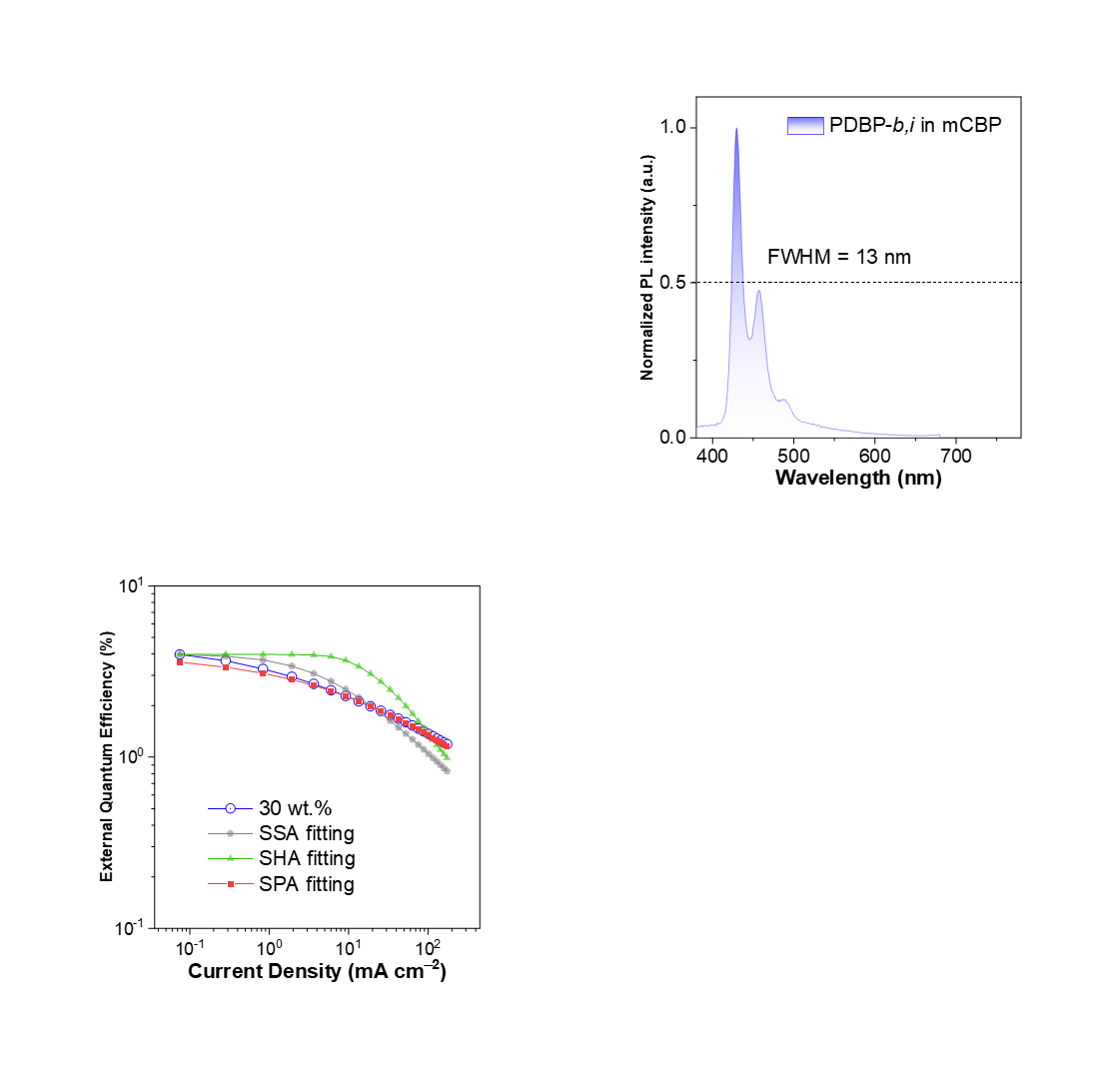


**Figure S29**. PL spectra for PBDP-*b,i* in doped film (in mCBP). The spectral narrowing mechanism of PDBP-*b,i* in host mCBP originated from aggregation-induced restriction of high-frequency vibration.

**
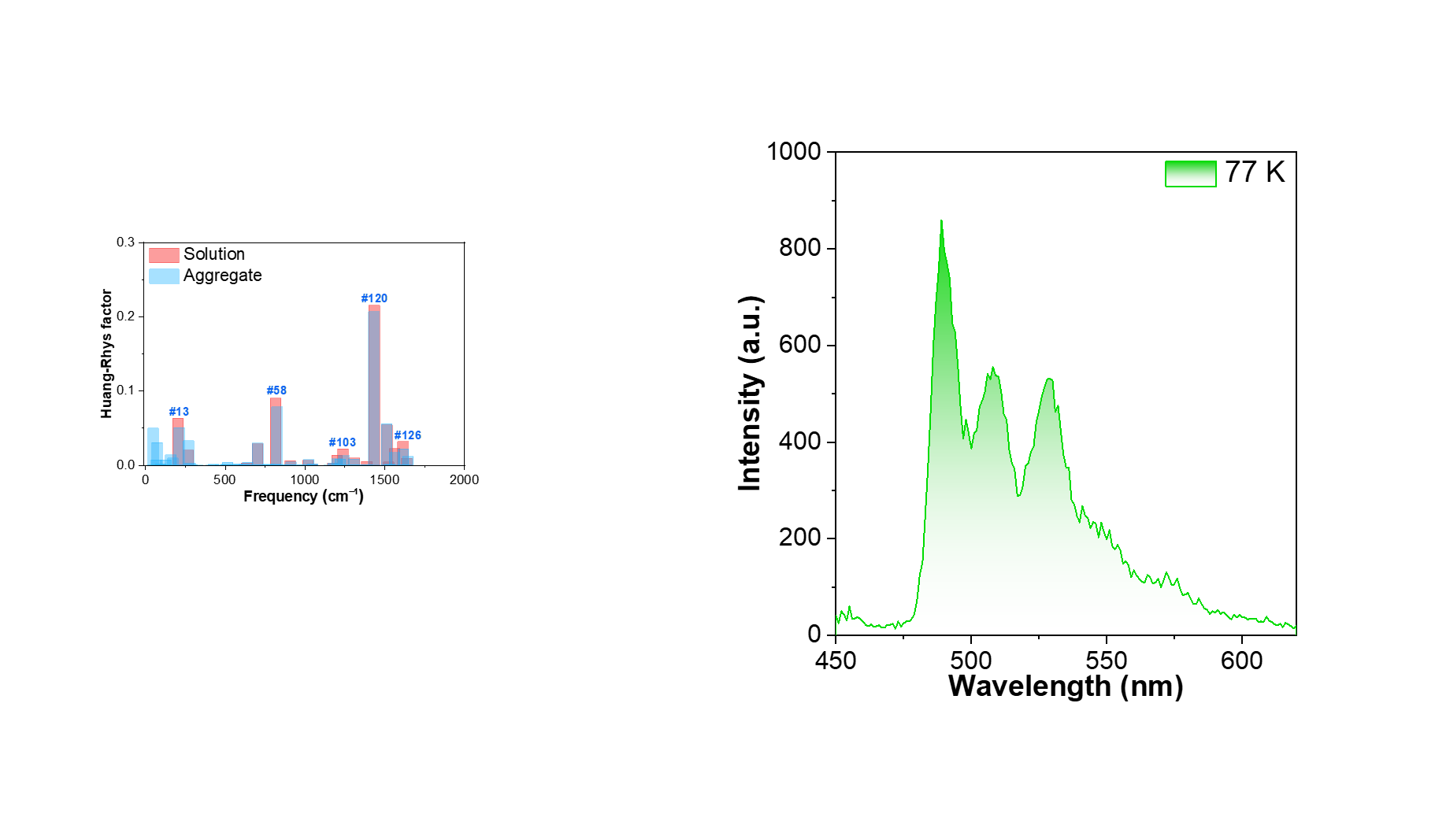
**

**Figure S30.** Low-temperature phospherescence spectrum in frozen toluene matrix (10^−5^ M) for PDBP-*b,i*.

**Table S5**. OLED performace of PDBP-*b*,*i*.

| **x**  **[wt%]** | **λ_EL_^a)^**  **[nm]** | **FWHM**  **[nm]** | ***V*_on_^b)^ [V]** | ***L*^c)^**  **[cd/m^2^]** | ***η*_c_^c)^**  **[cd/A]** | ***η*_p_^c)^**  **[lm/W]** | ***η*_ext_^d)^ max / @100 cd m^−2^ /**  **@1000 cd m^−2^ [%]** | **CIE (x, y)^e)^** |
| --- | --- | --- | --- | --- | --- | --- | --- | --- |
| 10 | 432 | 13 | 3.4 | 2152 | 1.79 | 1.65 | 3.99/2.05/1.20 | (0.160, 0.046) |
| 20 | 432 | 13 | 3.4 | 2392 | 1.88 | 1.73 | 4.20/2.20/1.18 | (0.156, 0.045) |
| 30 | 432 | 13 | 3.4 | 2700 | 2.05 | 1.90 | 3.98/2.28/1.22 | (0.158, 0.053) |

^a)^ 𝜆_EL_ = EL maximum; ^b)^ V_on_ = turn-on voltage at 1 cd m^−2^; ^c)^ Luminescence (L), current efficiency (𝜂_c_), power efficiency (𝜂_p_); ^d)^ external quantum efficiency (*η*_ext_); ^e)^ CIE = Commission Internationale de L’Eclairage, recorded at 10 mA cm^−2^.

**Singlet-singlet annihilation (SSA),^1^ singlet-heat annihilation (SHA)^1^ and singlet-polaron annihilation (SPA)^2^ fitting methods of PDBP-*b,i* based OLEDs.**

|  | $\frac{\eta_{SSA}}{\eta_{0}}=\frac{J_{0}}{4J}\left[ \sqrt{1+8\frac{J}{J_{0}}}-1 \right]$ | (1-1) |
| --- | --- | --- |
|  | $\frac{\eta_{SHA}}{\eta_{0}}=\frac{J_{0}}{4J}\left[ \sqrt{1+8\frac{J}{J_{0}}-2}\tau^{2}k_{sq}k_{th}J^{(1+\frac{1}{m})}\frac{1}{\alpha^{m}}-1 \right]$ | (1-2) |
|  | $\frac{\eta_{SPA}}{\eta_{0}}=\frac{1}{1+\left( \frac{J}{J_{0}} \right)^{\frac{1}{(l+1)}}}$ | (1-3) |

This is where $\eta_{0}$ is the external quantum efficiency without annihilation, and $J_{0}$ is the characteristics current density where $\eta_{SSA}$, $\eta_{SHA}$ and $\eta_{SPA}$ becomes half the initial $\eta_{0}$.


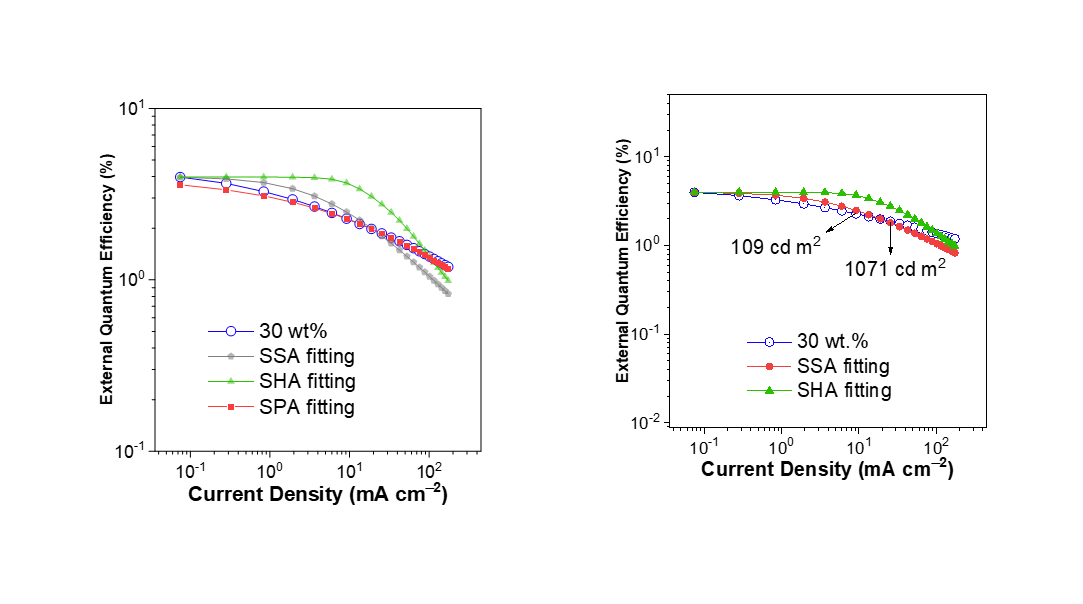


**Figure S31.** *η*_ext_−*J* characteristics of PDBP-*b,i* devices at doping concentration of 30 wt% with SSA, SHA and SPA models. Best fit was obtained with $J_{0}$ = 18.85 mA/cm^2^, *m =* 0.7, $\tau^{2}k_{sq}k_{th}$ = 1$\times$10^−10^, *l* = 1.5.

**Table S6**. Summary of the reported doped OLEDs with FWHM ≤ 25 nm.

| **Compound** | **Wavelength (nm)** | **FWHM (nm)** | **Doping concentration**  **(wt%)** | **Ref.** |
| --- | --- | --- | --- | --- |
| **PDBP-*b,i*** | 432 | 13 | 10/20/30 | **This work** |
| **TB-PB** | 482 | 11 | 1 | *Adv. Mater.* **2024**, *36*, 2410096. |
| ***ν-*DABNA** | 469 | 18 | 1 | *Nat. Photonics* **2019**, *13*, 678-682. |
| **m-v-DABNA** | 471 | 18 | 5 | *Chem. Eng. J.* **2022**, *432*, 134381. |
| **4F-v-DABNA** | 464 | 18 | 5 |  |
| **4F-m-v-DABNA** | 461 | 18 | 5 |  |
| **MesB-DIDOBNA-N** | 402 | 21 | 3 | *Adv. Mater.* **2023**, *35*, 2300997. |
| ***p*BP-DABNA-Me** | 464 | 23 | 5 | *Adv. Mter.* **2022**, *34*¸2207416. |
| **v-DABNA-Az1** | 459 | 19 | 2 | *Adv. Mater.* **2024**, *36*, 2402905. |
| **v-DABNA-Az2** | 458 | 17 | 2 |  |
| **v-DABNA-Az3** | 459 | 20 | 2 |  |
| **DPA-B3** | 450 | 15 | 1 | *Nat. Photonics,* **2024** *18*, 1161–1169. |
| **DPA-B4** | 457 | 14 | 1 |  |
| **pNAICZ-tPh** | 458 | 19 | 2 | *Adv. Mater.* **2025**, 2503839. |
| **CNBN** | 506 | 16 | 3 | *Angew. Chem. Int. Ed.* **2022**, *64*, e202500108. |
| **MCNBN** | 517 | 17 | 3 |  |
| **PCNBN** | 508 | 20 | 3 |  |
| **PMCNBN** | 519 | 22 | 3 |  |
| **BN3** | 456 | 18 | 1 | *Adv. Mater.* **2025**, 2502459. |
| **DBDS** | 456 | 18 | 1 |  |
| **DBDSe** | 456 | 18 | 1 |  |
| **BOBO-Z** | 445 | 18 | 1 | *Adv. Mater.* **2022,** 2107951. |
| **BOBS-Z** | 456 | 23 | 1 |  |
| **BSBS-Z** | 463 | 22 | 1 |  |
| **v-DABNA-OMe** | 465 | 23 | 1 | *Angew. Chem. Int. Ed.* **2021**, *60*, 17910-17914. |
| **BN2** | 467 | 23 | 1 | *Angew. Chem. Int. Ed.* **2022**, *61*, e202201588. |
| **BN3** | 458 | 23 | 1 |  |
| **3B-DPA** | 440 | 14 | 3 | *Angew. Chem. Int. Ed.* **2025**, e202503320. |
| **3B-DPA** | 438 | 15 | 3 |  |
| **4F-*v*-DABNA** | 464 | 18 | 5 | *Chem. Eng. J.* **2022**, *432*, 134381. |
| **4F-m-*v*-DABNA** | 461 | 18 | 5 |  |
| **CZ2CO** | 445 | 23 | 3 | *Angew. Chem. Int. Ed.* **2023**, *135*, e202215226. |
| **tBisICz** | 445 | 22 | 1 | *Adv. Sci.* **2021**, *8*, 2101137. |
| **tPBisICz** | 452 | 21 | 1 |  |
| **V-DABNA** | 458 | 17 | 1 | *Adv. Sci.* **2023**, *10*, 2205070. |
| **V-DABNA-F** | 477 | 15 | 1 |  |
| **DBCz-Mes** | 452 | 17 | 1 | *Sci. Adv.* **2023**, *9*, eadh1434. |
| **DOB2-DABNA-A** | 452 | 24 | 1 | *Nat. Commun.* **2024**, *15*, 2361. |
| **DOB2-DABNA-B-NP** | 471 | 23 | 1 |  |
| **DB-O** | 445 | 24 | 2 | *Adv. Mater.* **2024**, *36*, 2308314. |
| **DB-S** | 447 | 24 | 2 |  |
| **DB-SF1** | 464 | 24 | 5 | *Chem. Sci.* **2024**, *15*, 18076-18084. |
| **DB-SF2** | 460 | 22 | 5 |  |
| **[B-N]N2** | 441 | 20 | 1 | *Adv. Mater.* **2024**, *36*¸ 2409706. |
| **Cz-DBCz** | 487 | 19 | 1 | *Angew. Chem. Int. Ed.* **2023**, *62*, e202309923. |
| **Cz-DBTPA** | 481 | 18 | 1 |  |
| **DBNO** | 504 | 24 | 1 | *Angew. Chem. Int. Ed.* **2022**, *61*, e202200337. |
| **2** | 600 | 23 | 0.3 | *Angew. Chem. Int. Ed.* **2025**, *64*, e202500110. |
| **3** | 513 | 25 | 0.3 |  |
| **HBN** | 583 | 25 | 8 | *Angew. Chem. Int. Ed.* **2025**, *64*, e20241102. |
| **DBN-ICz** | 551 | 23 | 3 | *Adv. Mater.* **2023**, *35*, 2209396. |
| **DBN-bmICz** | 481 | 25 | 1 | *Angew. Chem. Int. Ed.* **2025**, *64*, e202504002. |
| **DBN-amICz** | 480 | 21 | 1 |  |
| **CNBN** | 506 | 16 | 3 | *Angew. Chem. Int. Ed.* **2025**, *64*, e202500108 |
| **PMCNBN** | 519 | 22 | 3 |  |
| **MCNBN** | 517 | 17 | 3 |  |
| **PCNBN** | 508 | 20 | 3 |  |
| **QB-DPA** | 502 | 17 | 1 | *Angew. Chem. Int. Ed.* **2025**, *64*, e202418348. |
| **QB-PXZ** | 516 | 20 | 1 |  |
| **BN-ICz-1** | 521 | 23 | 3 | *Angew. Chem. Int. Ed.* **2022**, *61*, e202202380. |
| **BN-ICz-2** | 521 | 23 | 3 |  |
| **tCzphB-Ph** | 527 | 24 | 2 | *Nat. Commun*. **2022**, *13*, 4876. |
| **v-DABNA-CN-Me** | 504 | 23 | 0.5 | *Adv. Mater.* **2022**, *34*, 2201778. |
| **DBT** | 520 | 24 | 2 | *Chem. Commun*. **2023**, *59*, 5126. |
| **ω-DABNA** | 512 | 25 | 0.5 | *J. Am. Chem. Soc.* **2023**, *145*, 1505. |
| **ω-DABNA-M** | 515 | 25 | 0.5 | *Nat. Commun*. **2024**, *15*, 3174. |
| **SF-BN1** | 460 | 23 | 3 | *Angew. Chem. Int. Ed.* **2025**, e202504723. |
| **DBNDS-TPh** | 525 | 19.2 | 5 | *Adv. Mater.* **2025**, *37*, 2416224. |
| **DBNDS-DFPh** | 523 | 19.8 | 2 |  |
| **DBNDS-CNPh** | 521 | 19.3 | 1 |  |
| **NT-2BN** | 517 | 21.5 | 1 | *Angew. Chem. Int. Ed*. **2024**, e202415113. |
| **PtN7N** | 518 | 24 | 6 | *Mater. Chem. Front.* **2018**, *2*, 704. |
| ***ββ*ICZ** | 508 | 19 | 1 | *Adv. Mater.* **2023**, *35*, 2211316. |
| ***ββ*CNICZ** | 509 | 19 | 1 |  |
| ***f-*DOABNA** | 445 | 24 | 1 | *Adv. Mater.* **2024**, *36*, 2402289. |
| **2tPAB** | 455 | 18 | 3 | *Org. Electronics* **2021**, *97*, 106275. |
| **3tPAB** | 451 | 20 | 3 |  |
| **BICz** | 442 | 18 | 3 | *Angew. Chem. Int. Ed.* **2025**, e202504002. |
| **BICz-1.5G** | 438 | 23 | 3 |  |
| **BICz-2G** | 447 | 23 | 3 |  |
| **NB-1** | 449 | 14 | 1 | *Nat. Photonics* **2024**, *23*, 519-526. |
| **NB-2** | 458 | 15 | 1 |  |
| **mMes_2_DICz** | 450 | 13 | 1 | *J. Mater. Chem. C.* **2022**, *10*, 7799-7802. |
| **tDIDCz** | 401 | 14 | 1 | *Small* **2020**, *16*, 1907569. |
| **m-FLDID** | 407 | 17 | 1 | *ACS Appl. Mater. Interfaces* **2021**, *13*, 14440-14446. |
| **tBisICz-PhCz** | 447 | 19 | 5 | *Adv. Mater.* **2023**, *10*, 2302619. |
| **Cz-DICz** | 460 | 18 | 4 | *Adv. Sci.* **2024**, *11*, 2307675. |
| **tDAmDIPz** | 458 | 17 | 1 | *Chem. Eng. J.* **2023**, *474*, |

**Table S7.** Summary of the reported high doping concentration (≥ 10 wt%) OLEDs with FWHM ≤ 40 nm.

| **Compound** | **Wavelength (nm)** | **FWHM (nm)** | **Doping concentration**  **(wt%)** | **Ref** |
| --- | --- | --- | --- | --- |
| **PDBP-*b,i*** | 432 | 13 | 10/20/30 | **This work** |
| ***t-*DABNA** | 465 | 26 | 10 | *Mater. Today Energy* **2021**, *21*, 100792. |
| ***t-*DAB-DPA** | 459 | 26 | 10 |  |
| **D-4SBN** | 500 | 31 | 10 | *Adv. Optical Mater*. **2024**, *12*, 2401754. |
| ***p*BP-DABNA-Me** | 464 | 23 | 10/15/20 | *Adv. Mater.* **2022**, *34*, 2207416. |
| **4tCz2B** | 508 | 26 | 10/20/30/50 | *Adv. Mater.* **2025**, 2500269. |
| **(*M*)-ABH-BNCz** | 495 | 30 | 10 | *Adv. Mater.* **2025**, 2420611. |
| **(*P*)-ABH-BNCz** | 495 | 30 | 10 |  |
| **BN-2PhC** | 492 | 32 | 10/15/20 | *Chem. Eng. J.* **2025**, *514*, 163327. |
| **BN-2TPA** | 492 | 30/32/32 | 10/15/20 |  |
| **BN-2DPXZ** | 490 | 38 | 10 |  |
| ***m-*PAz-BNCz** | 528 | 37 | 10/20 | *Adv. Mater.* **2025**, 2503383. |
| ***m-S*FAz-BNCz** | 532 | 38 | 10/20 |  |
| ***m*-DPAcPAz-BNCz** | 524 | 37 | 10/20 |  |
| ***m*-TPAz-BNCz** | 532 | 38 | 10/20 |  |
| **5Cz-BNO** | 467 | 38/39 | 10/20 | *Angew. Chem. Int. Ed.* **2025**, *64*, e202420489. |
| **5Cz-BN** | 502 | 29 | 10/20 |  |
| **4FICzBN** | 500 | 30 | 16 | *Adv. Opt. Mater.* **2024**, *12*, 2301217. |
| **SF1BN** | 496 | 27.6/27.8 | 10/15 | *Angew. Chem. Int. Ed.* **2022**, *61*, e202201886. |
| **SF3BN** | 500 | 34/37 | 10/15 |  |
| **D-Cz-BN** | 491 | 24 | 20 | *Angew. Chem. Int. Ed.* **2022**, *61*, e202113206. |
| **S-Cz508-BN** | 493/494 | 28/30 | 10/20 |  |
| **tCBNDADPO** | 468/472/472/472 | 24/28/28/28 | 10/20/30/40 | *Adv. Mater*. **2022**, *34*, 2110547. |
| **C-BN** | 452 | 28 | 12 | *Angew. Chem. Int. Ed.* **2022**, *134*, e202213585. |
| **BN-CP1** | 496 | 25 | 10/15/20/30 | *Adv. Mater.* **2022***, 34,* e2106954. |
| **BN-CP2** | 499/500/501/502 | 30/31/32/33 | 10/15/20/30 |  |
| **tDPAC-BN** | 460 | 26/28 | 10/20 | *Chem. Eng. J.* **2022**, *431*, 133221*.* |
| **tDMAC-BN** | 476 | 36 | 10/20 |  |
| **TCzBN-DPF** | 500/501 | 32/36 | 10/20 | *Mater. Horiz.* **2022**, *9*, 2226-2232. |
| **TCzBN-TMPh** | 488 | 27 | 10/20 |  |
| **TCzBN-oPh** | 496 | 29/31 | 10/20 |  |
| **NBNN2** | 538/539 | 38/39 | 10/20 | *Angew. Chem. Int. Ed.* **2023**, *62*, e202310943*.* |
| **SPAC-t-CzBN** | 480 | 24/25 | 10/20 | *Chem. Eng. J.* **2023***, 467,* 143557. |
| **SPBAC-tCzBN** | 480 | 25 | 10/20 |  |
| **o-SPAC-tCzBN** | 492 | 26/28 | 10/20 |  |
| **p-1-PCzBN** | 497/497/498 | 27 | 12/20/30 | *Adv. Opt. Mater.* **2023***, 11*, 2203002. |
| **m-1-PCzBN** | 503/505/508 | 31/33/34 | 12/20/30 |  |
| **IDAD-BNCz** | 494/496/498 | 29/31/34 | 10/15/20 | *Angew. Chem. Int. Ed.* **2024**, *63*, e202401120. |
| **TIDAD-BNCz** | 496/496/498 | 34/34/36 | 10/15/20 |  |
| **Endo-D1** | 479 | 35 | 10/15/20 | *Angew. Chem. Int. Ed.* **2024**, e202415607. |
| **Endo-D2** | 472 | 38 | 10/15/20 |  |
| **DtCzBN-CNBT1** | 508 | 38 | 15 | *Adv. Mater.* **2024**, e2412761. |
| **DtCzB**  **N-CNB**  **T2** | 508 | 37 | 15 |  |
| **BNSi** | 484 | 27/27/28 | 10/15/20 | *J. Mater. Chem. C* **2024**, *12*, 18725-18731. |
| **BNNGe** | 494/495/495/496 | 25/26/26/27 | 10/15/20/30 | *Adv. Opt. Mater.* **2024**, *12*, 2401033. |
| **BNOGe** | 464 | 37 | 10 |  |
| **BNSGe** | 488/488/488/489 | 32/32/33/34 | 10/15/20/30 |  |
| **BN-MP-Cz** | 470/470 | 28/30 | 10/20 | *Aggregate* **2024**, *5*, e585. |
| **2BN-MP-CmCP** | 486/486 | 26/27 | 10/20 |  |
| **tCzBN-PQCz** | 517 | 38 | 10 | *Chem. Eng. J.* **2024**, *481*, 148794*.* |
| **IPrBN** | 459 | 22 | 10 | *Adv. Funct. Mater.* **2024**, *35*, 2414635. |
| **IPrBN-mCP** | 451 | 20 | 10 |  |
| **BN-TB** | 496/496/496 | 32/34/34 | 10/20/30 | *Chem. Eng. J.* **2025**, *515*, 163725. |

**Table S8**. Crystal data and structure refinements for PDBP-*a*,*i* and PDBP-*b*,*i*.

| **Identification code** | **PDBP-*a,i*** | **PDBP-*b,i*** |
| --- | --- | --- |
| **CCDC** | 2444441 | 2444438 |
| **Empirical formula** | C_32_H_22_N_2_ | C_16_H_11_N |
| **Formula weight** | 434.51 | 217.26 |
| **Temperature/K** | 149.99(10) | 150.00(10) |
| **Crystal system** | monoclinic | tetragonal |
| **Space group** | Cc | P4_2_/n |
| **a/Å** | 9.26042(17) | 15.47206(9) |
| **b/Å** | 18.9045(3) | 15.47206(9) |
| **c/Å** | 13.2996(3) | 9.62327(8) |
| ***α*/°** | 90 | 90 |
| ***β*/°** | 106.413(2) | 90 |
| ***γ*/°** | 90 | 90 |
| **Volume/Å^3^** | 2233.40(8) | 2303.66(3) |
| **Z** | 4 | 8 |
| ***ρ*_calc_g/cm^3^** | 1.292 | 1.253 |
| ***μ*/mm^‑1^** | 0.580 | 0.562 |
| **F(000)** | 912.0 | 912.0 |
| **Crystal size/mm^3^** | 0.1 × 0.05 × 0.05 | 0.1 × 0.05 × 0.02 |
| **Radiation** | CuK*α* (*λ* = 1.54184) | CuKα (λ = 1.54184) |
| **2*Θ* range for data collection/°** | 9.356 to 153.196 | 8.082 to 153.328 |
| **Index ranges** | -11 ≤ h ≤ 8, -23 ≤ k ≤ 22, -16 ≤ l ≤ 16 | -19 ≤ h ≤ 19, -19 ≤ k ≤ 18, -11 ≤ l ≤ 12 |
| **Reflections collected** | 20835 | 29152 |
| **Independent reflections** | 4094 [R_int_= 0.0419, R_sigma_= 0.0320] | 2411 [R_int_= 0.0288, R_sigma_= 0.0143] |
| **Data/restraints/parameters** | 4094/2/307 | 2411/0/154 |
| **Goodness-of-fit on F^2^** | 1.049 | 1.139 |
| **Final R indexes [I>=2σ (I)]** | R_1_= 0.0325, wR_2_= 0.0795 | R_1_= 0.0438, wR_2_= 0.1279 |
| **Final R indexes [all data]** | R_1_= 0.0354, wR_2_= 0.0816 | R_1_= 0.0467, wR_2_= 0.1303 |
| **Largest diff. peak/hole / e Å^-3^** | 0.12/-0.16 | 0.22/-0.26 |
| **Flack parameter** | 0.3(3) |  |

**Table S9**. Bond lengths for PDBP-*a,i*.

| **Atom** | **Atom** | **Length/Å** | **Atom** | **Atom** | **Length/Å** |
| --- | --- | --- | --- | --- | --- |
| **N1** | **C20** | 1.432(3) | **C10** | **C9** | 1.422(3) |
| **N1** | **C1** | 1.435(3) | **C16** | **C17** | 1.367(4) |
| **N1** | **C21** | 1.423(3) | **C16** | **C15** | 1.405(4) |
| **C13** | **C12** | 1.414(3) | **C3** | **C4** | 1.369(3) |
| **C13** | **C18** | 1.418(3) | **C4** | **C5** | 1.411(4) |
| **C13** | **C14** | 1.418(3) | **C15** | **C14** | 1.359(3) |
| **N2** | **C10** | 1.410(3) | **C27** | **C28** | 1.379(3) |
| **N2** | **C27** | 1.440(3) | **C27** | **C32** | 1.390(3) |
| **N2** | **C11** | 1.411(3) | **C5** | **C6** | 1.363(4) |
| **C12** | **C11** | 1.375(3) | **C9** | **C8** | 1.362(3) |
| **C2** | **C7** | 1.422(3) | **C24** | **C25** | 1.385(4) |
| **C2** | **C1** | 1.417(3) | **C24** | **C23** | 1.383(4) |
| **C2** | **C3** | 1.414(3) | **C28** | **C29** | 1.389(3) |
| **C18** | **C19** | 1.414(3) | **C25** | **C26** | 1.386(3) |
| **C18** | **C17** | 1.416(3) | **C22** | **C21** | 1.402(3) |
| **C20** | **C19** | 1.365(3) | **C22** | **C23** | 1.382(3) |
| **C20** | **C11** | 1.427(3) | **C21** | **C26** | 1.395(3) |
| **C7** | **C8** | 1.415(3) | **C32** | **C31** | 1.385(4) |
| **C7** | **C6** | 1.420(3) | **C29** | **C30** | 1.378(5) |
| **C1** | **C10** | 1.378(3) | **C30** | **C31** | 1.382(5) |

**Table S10**. Bond angles for PDBP-*a,i*.

| **Atom** | **Atom** | **Atom** | **Angle/˚** | **Atom** | **Atom** | **Atom** | **Angle/˚** |
| --- | --- | --- | --- | --- | --- | --- | --- |
| **C20** | **N1** | **C1** | 111.58(16) | **C17** | **C16** | **C15** | 120.0(2) |
| **C21** | **N1** | **C20** | 117.91(17) | **C4** | **C3** | **C2** | 120.8(2) |
| **C21** | **N1** | **C1** | 116.92(17) | **C3** | **C4** | **C5** | 120.5(2) |
| **C12** | **C13** | **C18** | 119.78(19) | **C16** | **C17** | **C18** | 120.7(2) |
| **C12** | **C13** | **C14** | 121.4(2) | **C14** | **C15** | **C16** | 121.0(2) |
| **C18** | **C13** | **C14** | 118.8(2) | **C28** | **C27** | **N2** | 119.17(19) |
| **C10** | **N2** | **C27** | 120.34(17) | **C28** | **C27** | **C32** | 120.4(2) |
| **C10** | **N2** | **C11** | 116.64(17) | **C32** | **C27** | **N2** | 120.4(2) |
| **C11** | **N2** | **C27** | 116.72(17) | **C15** | **C14** | **C13** | 120.5(2) |
| **C11** | **C12** | **C13** | 120.5(2) | **N2** | **C11** | **C20** | 117.22(17) |
| **C1** | **C2** | **C7** | 118.5(2) | **C12** | **C11** | **N2** | 123.42(19) |
| **C3** | **C2** | **C7** | 118.7(2) | **C12** | **C11** | **C20** | 119.35(18) |
| **C3** | **C2** | **C1** | 122.78(19) | **C6** | **C5** | **C4** | 120.1(2) |
| **C19** | **C18** | **C13** | 118.57(19) | **C8** | **C9** | **C10** | 120.1(2) |
| **C19** | **C18** | **C17** | 122.4(2) | **C23** | **C24** | **C25** | 118.9(2) |
| **C17** | **C18** | **C13** | 119.0(2) | **C27** | **C28** | **C29** | 119.5(2) |
| **C19** | **C20** | **N1** | 122.20(19) | **C24** | **C25** | **C26** | 120.8(2) |
| **C19** | **C20** | **C11** | 120.50(19) | **C23** | **C22** | **C21** | 120.2(2) |
| **C11** | **C20** | **N1** | 117.29(17) | **C22** | **C21** | **N1** | 120.65(19) |
| **C8** | **C7** | **C2** | 118.6(2) | **C26** | **C21** | **N1** | 120.80(18) |
| **C8** | **C7** | **C6** | 122.4(2) | **C26** | **C21** | **C22** | 118.5(2) |
| **C6** | **C7** | **C2** | 119.0(2) | **C9** | **C8** | **C7** | 121.7(2) |
| **C2** | **C1** | **N1** | 120.12(18) | **C31** | **C32** | **C27** | 119.9(2) |
| **C10** | **C1** | **N1** | 118.19(19) | **C30** | **C29** | **C28** | 120.1(3) |
| **C10** | **C1** | **C2** | 121.64(19) | **C29** | **C30** | **C31** | 120.5(3) |
| **C20** | **C19** | **C18** | 120.9(2) | **C25** | **C26** | **C21** | 120.4(2) |
| **N2** | **C10** | **C9** | 122.4(2) | **C22** | **C23** | **C24** | 121.1(2) |
| **C1** | **C10** | **N2** | 118.53(18) | **C30** | **C31** | **C32** | 119.6(3) |
| **C1** | **C10** | **C9** | 119.0(2) | **C5** | **C6** | **C7** | 120.9(2) |

**Table S11**. Bond lengths for PDBP-*b,i*.

| **Atom** | **Atom** | **Length/Å** | **Atom** | **Atom** | **Length/Å** |
| --- | --- | --- | --- | --- | --- |
| **N1** | **C2** | 1.4011(15) | **C5** | **C10** | 1.4135(17) |
| **N1** | **C4** | 1.3996(15) | **C7** | **C4^1^** | 1.3764(16) |
| **N1** | **C9** | 1.4385(14) | **C8** | **C11** | 1.3726(18) |
| **C2** | **C4^1^** | 1.4368(15) | **C9** | **C14** | 1.3818(15) |
| **C2** | **C6** | 1.3741(17) | **C9** | **C16** | 1.3824(16) |
| **C3** | **C5** | 1.4213(16) | **C10** | **C12** | 1.3725(17) |
| **C3** | **C7** | 1.4165(17) | **C11** | **C12** | 1.4092(17) |
| **C3** | **C8** | 1.4140(16) | **C13** | **C14** | 1.3898(16) |
| **C4** | **C2^1^** | 1.4369(15) | **C13** | **C15** | 1.3818(18) |
| **C4** | **C7^1^** | 1.3764(16) | **C15** | **C17** | 1.3897(18) |
| **C5** | **C6** | 1.4206(16) | **C16** | **C17** | 1.3850(16) |

Symmetry code: ^1^3/2-X,1/2-Y,+Z

**Table S12**. Bond angles for PDBP-*b,i.*

| **Atom** | **Atom** | **Atom** | **Angle/˚** | **Atom** | **Atom** | **Atom** | **Angle/˚** |
| --- | --- | --- | --- | --- | --- | --- | --- |
| **C2** | **N1** | **C9** | 118.76(10) | **C2** | **C6** | **C5** | 121.69(11) |
| **C4** | **N1** | **C2** | 122.86(10) | **C4^1^** | **C7** | **C3** | 121.87(11) |
| **C4** | **N1** | **C9** | 118.04(9) | **C11** | **C8** | **C3** | 121.15(11) |
| **N1** | **C2** | **C4^1^** | 118.37(11) | **C14** | **C9** | **N1** | 119.67(10) |
| **C6** | **C2** | **N1** | 122.09(10) | **C14** | **C9** | **C16** | 121.17(10) |
| **C6** | **C2** | **C4^1^** | 119.53(11) | **C16** | **C9** | **N1** | 119.15(10) |
| **C7** | **C3** | **C5** | 118.80(11) | **C12** | **C10** | **C5** | 121.15(11) |
| **C8** | **C3** | **C5** | 118.83(11) | **C8** | **C11** | **C12** | 119.98(11) |
| **C8** | **C3** | **C7** | 122.37(11) | **C10** | **C12** | **C11** | 120.10(11) |
| **N1** | **C4** | **C2^1^** | 118.66(10) | **C15** | **C13** | **C14** | 120.17(10) |
| **C7^1^** | **C4** | **N1** | 122.04(10) | **C9** | **C14** | **C13** | 119.24(10) |
| **C7^1^** | **C4** | **C2^1^** | 119.30(11) | **C13** | **C15** | **C17** | 119.96(11) |
| **C6** | **C5** | **C3** | 118.79(11) | **C9** | **C16** | **C17** | 119.25(11) |
| **C10** | **C5** | **C3** | 118.77(11) | **C16** | **C17** | **C15** | 120.20(12) |
| **C10** | **C5** | **C6** | 122.44(11) |  |  |  |  |

Symmetry code: ^1^3/2-X,1/2-Y,+Z

**References**

[1] Nakanotani, H; Sasabe, H. & Adachi, C. Singlet-singlet and singlet-heat annihilations in fluorescence-based organic light-emitting diodes under steady-state high current density. *Appl. Phys. Lett.* **86**, 213506 (2005).

[2] Kuwae, H. et al. Suppression of external quantum efficiency roll-off of nanopatterned organic-light emitting diodes at high current densities. *J. Appl. Phys.* **18**, 155501 (2015).
